# Simultaneous inpainting and denoising by directional global three-part decomposition: connecting variational and Fourier domain-based image processing

**DOI:** 10.1098/rsos.171176

**Published:** 2018-07-25

**Authors:** D. H. Thai, C. Gottschlich

**Affiliations:** 1 Department of Statistical Science, Duke University, Box 90251, Durham, NC 27708-0251, USA; 2 Institute for Mathematical Stochastics, University of Goettingen, Goldschmidtstrasse 7, 37077 Göttingen, Germany

**Keywords:** image decomposition, inverse problem, image inpainting, image denoising, textureimage, feature extraction

## Abstract

We consider the very challenging task of restoring images (i) that have a large number of missing pixels, (ii) whose existing pixels are corrupted by noise, and (iii) that ideally contain both cartoon and texture elements. The combination of these three properties makes this inverse problem a very difficult one. The solution proposed in this manuscript is based on directional global three-part decomposition (DG3PD) (Thai, Gottschlich. 2016 *EURASIP. J. Image Video Process.* **2016**, 1–20 (doi:10.1186/s13640-015-0097-y)) with a directional total variation norm, directional G-norm and ℓ_∞_-norm in the curvelet domain as key ingredients of the model. Image decomposition by DG3PD enables a decoupled inpainting and denoising of the cartoon and texture components. A comparison with existing approaches for inpainting and denoising shows the advantages of the proposed method. Moreover, we regard the image restoration problem from the viewpoint of a Bayesian framework and we discuss the connections between the proposed solution by function space and related image representation by harmonic analysis and pyramid decomposition.

## 1. Introduction and related work

Image enhancement and image restoration are two superordinate concepts in image processing which encompass a plethora of methods to solve a multitude of important real-world problems [1,2]. Image enhancement has the goal of improving an input image for a specific application, e.g. in areas such as medical image processing, biometric recognition, computer vision, optical character recognition, texture recognition or machine inspection of surfaces [3–5]. Methods for image enhancement can be grouped by the domain in which they perform their operations: images are processed in the spatial domain or Fourier domain, or modified, e.g. in the wavelet or curvelet domain [6]. The types of enhancement methods include contextual filtering, e.g. for fingerprint image enhancement [7–9], contrast enhancement, e.g. by histogram equalization [10], and image super-resolution [11]. Image restoration is connected to the notion that a given input image suffers from degradation and the goal is restore an ideal version of it. Degradations are caused by various types of noise, missing pixels or blurring and their countermeasures are denoising, inpainting and deblurring. In general, one has to solve a linear or nonlinear inverse problem to reconstruct the ideal image from its given degraded version. Denoising aims to remove noise from an image and denoising methods include total variation (TV) minimization-based approaches [12], the application of non-local means (NL means) [13] or other dictionaries of image patches for smoothing, and adaptive thresholding in the wavelet domain [14]. Inpainting [15] is the filling-in of missing pixels from the available information in the image, and it is applied for scratch removal from scanned photographs, for occlusion filling, for removing objects or persons from images (in image forgery [16] or for special effects), and for filling-in of pixels which were lost during the transmission of an image or left out on purpose for image compression [17]. Deblurring [18] addresses the removal of blurring artefacts and is not the focus of this paper.

Rudin *et al.* [19] pioneered two-part image decomposition by TV regularization for denoising. Shen & Chan [20] applied TV regularization to image inpainting, called the TV inpainting model, and they also suggested image inpainting by curvature-driven diffusions [21]. Starck *et al.* [22] defined a model for two-part decomposition based on the dictionary approach. Then, Elad *et al.* [23] applied this decomposition idea for image inpainting by introducing the indicator function in the ℓ_2_ norm of the residual; see eqn (6) in [23]. Esedoglu & Shen [24] introduced two inpainting models based on the Mumford–Shah model [25] and its higher order correction—the Mumford–Shah–Euler image model. They also presented numerical computation based on the *Γ*-convergence approximations [26,27]. Shen *et al.* [28] proposed image inpainting based on bounded variation and elastica models for non-textured images.

Image inpainting can be an easy or difficult problem depending on the amount of missing pixels [21], the complexity of the image content and whether prior knowledge about the image content is available. Methods have been proposed which perform only cartoon inpainting (also referred to as structure inpainting) [20,28,29] or only texture inpainting [30]. Images which consist of both cartoon (structure) and texture components are more challenging to inpaint. Bertalmio *et al.* [31], Elad *et al.* [23] and Cai *et al.* [32] have proposed methods for inpainting which can handle images with both cartoon (structure) and texture components.

In this paper, we tackle an even more challenging problem. Consider an input image **f** which has the following three properties:

(i) a large percentage of pixels in **f** are missing and shall be inpainted,
(ii) the known pixels in **f** are corrupted by noise,
(iii) **f** contains both cartoon and texture elements.

The co-occurrence of noise and missing pixels in an image with cartoon and texture components increases the difficulty of both the inpainting problem and the denoising problem. A multitude of methods have been proposed for inpainting and denoising. Existing inpainting methods in the literature typically assume that the non-missing pixels in a given image contain only a small amount of noise or are noise free, and existing methods for denoising typically assume that all pixels of the noisy image are known. The proposed method for solving this challenging problem is inspired by the works of Efros & Leung [30], Bertalmio *et al.* [31], Vese & Osher [33], Aujol & Chambolle [34], Buades *et al.* [13] and Elad *et al.* [23], and is based on the directional global three-part decomposition (DG3PD) [35]. The DG3PD method decomposes an image into three parts: a cartoon image, a texture image and a residual image. Advantages of the DG3PD model lie in the properties which are enforced on the cartoon and texture images. The geometric objects in the cartoon image have a very smooth surface and sharp edges. The texture image yields oscillating patterns on a defined scale which is both smooth and sparse. Recently, the texture images have been applied as a very useful feature for fingerprint segmentation [35–37].

**Figure 1.**
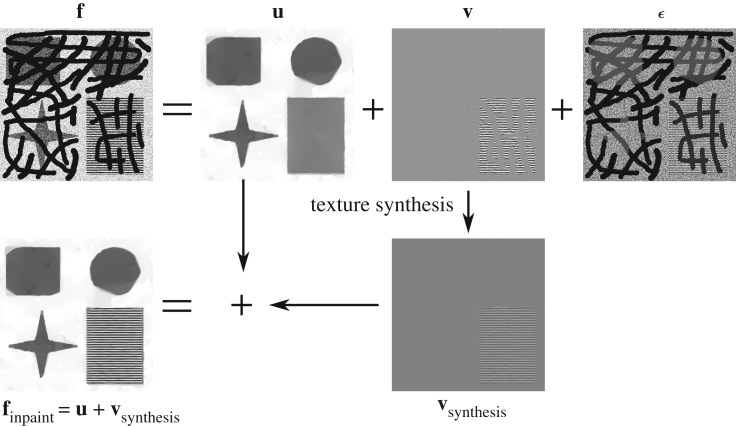
Overview over the DG3PD image inpainting and denoising process.

We address the challenging task of simultaneous inpainting and denoising in the following way. The advanced DG3PD model introduced in the next section decomposes a noisy input image **f** (with missing regions *D*) into cartoon **u**, texture **v** and residual ***ϵ*** components. At the same time, the missing regions *D* of the cartoon component **u** are interpolated and the available regions of **u** are denoised by the advantage of multi-directional bounded variation. This effect benefits from the help of the indicator function in the measurement of the residual, i.e. $∥C\{\chi_{D}^{c}\cdot^{\times }\epsilon \}{∥}_{\ell_{\infty }}$ in (2.1). However, texture **v** is not interpolated due to the ‘cancelling’ effect of this supremum norm for residual in unknown regions. Therefore, the texture component **v** is inpainted and denoised by a dictionary-based approach instead. The DG3PD decomposition drives noise into the residual component ***ϵ***, which is discarded. The reconstruction of the ideal version of **f** is obtained by summation of the inpainted and denoised cartoon and texture components (see figure 1 for a visual overview).

Moreover, we uncover the link between the calculus of variations [38–40] and filtering in the Fourier domain [41] by analysing the solution of the convex minimization in equation (2.1), i.e. roughly speaking the solution of the DG3PD inpainting model which can be understood as the response of the lowpass filter $\hat{\mathrm{LP}}(\omega )$, highpass filter $\hat{\mathrm{HP}}(\omega )$ and bandpass filter $\hat{\mathrm{BP}}(\omega ),$ and the unity condition is satisfied, i.e.

$$\hat{\mathrm{LP}}(\omega )+\hat{\mathrm{BP}}(\omega )+\hat{\mathrm{HP}}(\omega )=1,$$

where ***ω***∈[ − *π*, *π*]^2^ is a coordinator in the Fourier domain. We observe that this decomposition is similar to the wavelet or pyramidal decomposition scheme [42–44]. However, the basis elements obtaining the decomposition, i.e. the scaling function and frame (or wavelet-like) function, are constructed by discrete differential operators (due to the discrete setting in minimizing (2.1)), which are referred to as wavelet-like operators in [45]. In particular,

— the scaling function and wavelet-like function for the cartoon **u** are from the effect of the multi-directional TV norm,
— the scaling function and wavelet-like function to extract the texture **v** are reconstructed by the effect of the multi-directional G-norm,
— the effect of the ℓ_∞_ norm $∥C\{\chi_{D}^{c}\cdot^{\times }\epsilon \}{∥}_{\ell_{\infty }}$ is to remove the remaining signal in the known regions of the residual ***ϵ*** (due to the duality property of ℓ_∞_).

We also describe flowcharts to show that the method of variational calculus (or the DG3PD inpainting) is a closed loop pyramidal decomposition, which is different from an open loop one, e.g. wavelet [46], curvelet [47], see §7. By numerics, we observe that the closed loop filter design by the calculus of variation will result in lowpass, highpass and bandpass filters which are ‘unique’ for different images (figure 17). We also analyse the DG3PD inpainting model from the perspective of a Bayesian framework and then define a discrete innovation model for this inverse problem.

This paper is organized as follows. In §2, we describe the DG3PD model for image inpainting and denoising. In §3, we show how to compute the solution of the convex minimization in the DG3PD inpainting problem by the augmented Lagrangian method. In §4, we describe the proposed method for texture inpainting and denoising. In §5, we compare the proposed method with existing ones (TVL2 inpainting [48,49]). Moreover, in order to enhance our evaluation in terms of simultaneous inpainting and denoising effects, we also perform a comparison with the NL-means filter for denoising, namely block-matching and three-dimensional filtering (BM3D), in [50]. In §6, we consider our inverse problem from a statistical point of view, i.e. the Bayesian framework, to describe how to select priors for cartoon **u** and texture **v**. We analyse the relation between the calculus of variations and the traditional pyramid decomposition scheme, e.g. Gaussian pyramid, in §7. We conclude the study with §8. For more detailed notation and mathematical preliminaries, we refer the reader to [36,51].

## 2. Inpainting by DG3PD

We define a method for restoration of the original noisy/non-noisy image **f** with a set of known missing regions *D*. The proposed model is a generalized version of DG3PD [35] for the inpainting and denoising problem. This modification for our DG3PD inpainting is inspired by Elad *et al.* [23].

In particular, the discrete image **f** (size *m* × *n*) with missing regions *D* is simultaneously decomposed into cartoon **u**, texture **v** and noise ***ϵ***; in particular, cartoon **u** is interpolated on the missing regions due to the indicator function *χ*_*D*_ in the modified model.

A set of missing regions *D* on a bounded domain *Ω* is defined by the indicator function *χ*_*D*_ whose complement is

$$\chi_{D}^{c}[k]=\left\{ \begin{array}{ll} 1, & k\in \Omega \setminus D,\\ 0, & k\in D. \end{array}\right.$$

Instead of putting *χ*^*c*^_*D*_ on the ℓ_2_ norm of the residual (see eqns (4) and (6) in [23]), we introduce *χ*^*c*^_*D*_ for the residual in the DG3PD model with the point-wise multiplication operator · × , i.e. $∥C\{\chi_{D}^{c}\cdot^{\times }\epsilon \}{∥}_{\ell_{\infty }}$. We propose the DG3PD inpainting as follows:

2.1 $$\begin{array}{rl} \left(u^{*},v^{*},\epsilon^{*},g^{*}\right) & =\underset{(u,v,\epsilon ,g)\in X^{3+S}}{\arg\;\min}\{\underset{:=∥\nabla_{L}^{+}u{∥}_{\ell_{1}}}{\underbrace{\sum_{l=0}^{L-1}{∥\cos\left(\frac{\pi l}{L}\right)uD_{n}^{T}+\sin\left(\frac{\pi l}{L}\right)D_{m}u∥}_{\ell_{1}}}}\\ & \;+\mu_{1}\underset{:=∥v{∥}_{G_{S}}}{\underbrace{\sum_{s=0}^{S-1}∥g_{s}{∥}_{\ell_{1}}}}+\mu_{2}∥v{∥}_{\ell_{1}},\\ s.t.{∥C\{\chi_{D}^{c}\cdot^{\times }\epsilon \}∥}_{\ell_{\infty }} & \leq \nu ,\;v=\underset{={\mathrm{div}}_{S}^{+}g}{\underbrace{\sum_{s=0}^{S-1}\left[\cos\left(\frac{\pi s}{S}\right)g_{s}D_{n}^{T}+\sin\left(\frac{\pi s}{S}\right)D_{m}g_{s}\right]}},\;f=u+v+\epsilon ,\} \end{array}$$

where C is a curvelet transform [47], **g**_*s*_ is an unknown variable in the *G*-norm [52] and a finite difference matrix is

$$D_{m}=\left(\begin{array}{ccccc} -1 & 1 & 0 & \ldots & 0\\ 0 & -1 & 1 & \ldots & 0\\ \vdots & \vdots & \vdots & \ddots & \vdots \\ 0 & 0 & 0 & \ldots & 1\\ 1 & 0 & 0 & \ldots & -1 \end{array}\right)\in R^{m\times m}$$

and $D_{n}\in R^{n\times n}$ is similar.

Note that if there are no missing regions, i.e. *χ*^*c*^_*D*_[***k***] = 1, ∀***k***∈*Ω*, the minimization problem (2.1) becomes the DG3PD model [35]. Next, we discuss how to solve the DG3PD-inpainting model (2.1).

## 3. Solution of the DG3PD inpainting model

In this section, we present a numerical algorithm for obtaining the solution of the DG3PD-inpainting model stated in (2.1). To simplify the calculation, we introduce three new variables

$$\begin{array}{l} r_{b}=\cos\left(\frac{\pi b}{L}\right)uD_{n}^{T}+\sin\left(\frac{\pi b}{L}\right)D_{m}u,\;b=0,\ldots ,L-1,\\ w_{a}=g_{a},\;a=0,\ldots ,S-1,\\ e=\chi_{D}^{c}\cdot^{\times }\epsilon \end{array}$$

and denote G*(**e**/*ν*) as the indicator function on the feasible convex set *A*(*ν*) of (2.1), i.e.

$$A(\nu )=\{e\in X:\;∥C\{e\}{∥}_{\ell_{\infty }}\leq \nu \}\;\mathrm{and}\;G^{*}\left(\frac{e}{\nu }\right)=\left\{ \begin{array}{ll} 0, & \epsilon \in A(\nu ),\\ +\infty , & \mathrm{else}. \end{array}\right.$$

Owing to the constrained minimization problem in (2.1), the augmented Lagrangian method is applied to turn it into an unconstrained one as

3.1 $$\min_{(u,v,\epsilon ,e,{\left[r_{l}\right]}_{l=0}^{L-1},{\left[w_{s}\right]}_{s=0}^{S-1},{\left[g_{s}\right]}_{s=0}^{S-1})\in X^{L+2S+4}}L(\cdot ;{\left[\lambda_{1l}\right]}_{l=0}^{L-1},{\left[\lambda_{2s}\right]}_{s=0}^{S-1},\lambda_{3},\lambda_{4},\lambda_{5}),$$

where the Lagrange function is

$$\begin{array}{rl} L(\cdot \;;\cdot ) & =\sum_{l=0}^{L-1}∥r_{l}{∥}_{\ell_{1}}+\mu_{1}\sum_{s=0}^{S-1}∥w_{s}{∥}_{\ell_{1}}+\mu_{2}{∥v∥}_{\ell_{1}}+G^{*}\left(\frac{e}{\nu }\right)\\ & \;+\frac{\beta_{1}}{2}\sum_{l=0}^{L-1}{∥r_{l}-\cos\left(\frac{\pi l}{L}\right)uD_{n}^{T}-\sin\left(\frac{\pi l}{L}\right)D_{m}u+\frac{\lambda_{1l}}{\beta_{1}}∥}_{\ell_{2}}^{2}\\ & \;+\frac{\beta_{2}}{2}\sum_{s=0}^{S-1}{∥w_{s}-g_{s}+\frac{\lambda_{2s}}{\beta_{2}}∥}_{\ell_{2}}^{2}+\frac{\beta_{3}}{2}\\ & \;{∥v-\sum_{s=0}^{S-1}\left[\cos\left(\frac{\pi s}{S}\right)g_{s}D_{n}^{T}+\sin\left(\frac{\pi s}{S}\right)D_{m}g_{s}\right]+\frac{\lambda_{3}}{\beta_{3}}∥}_{\ell_{2}}^{2}\\ & \;+\frac{\beta_{4}}{2}{∥f-u-v-\epsilon +\frac{\lambda_{4}}{\beta_{4}}∥}_{\ell_{2}}^{2}+\frac{\beta_{5}}{2}{∥e-\chi_{D}^{c}\cdot^{\times }\epsilon +\frac{\lambda_{5}}{\beta_{5}}∥}_{\ell_{2}}^{2}. \end{array}$$

Similar to [35], the alternating directional method of multipliers is applied to solve the unconstrained minimization problem (3.1). Its minimizer is numerically computed through iterations, *t* = 1, 2, …,

3.2 $$\begin{array}{rl} & \left(u^{\left(t\right)},v^{\left(t\right)},\epsilon^{\left(t\right)},e^{\left(t\right)},{\left[r_{l}^{\left(t\right)}\right]}_{l=0}^{L-1},{\left[w_{s}^{\left(t\right)}\right]}_{s=0}^{S-1},{\left[g_{s}^{\left(t\right)}\right]}_{s=0}^{S-1}\right)\\ & \;=\arg\min L(u,v,\epsilon ,e,{\left[r_{l}\right]}_{l=0}^{L-1},{\left[w_{s}\right]}_{s=0}^{S-1},{\left[g_{s}\right]}_{s=0}^{S-1};\;{\left[\lambda_{1l}^{\left(t-1\right)}\right]}_{l=0}^{L-1},{\left[\lambda_{2s}^{\left(t-1\right)}\right]}_{s=0}^{S-1},\lambda_{3}^{\left(t-1\right)},\lambda_{4}^{\left(t-1\right)},\lambda_{5}^{\left(t-1\right)}), \end{array}$$

and the Lagrange multipliers are updated after every step *t*. We also initialize **u**^(0)^ = **f**, **v**^(0)^ = ***ϵ***^(0)^ = **e**^(0)^ = [**r**^(0)^_*l*_]^*L*−1^_*l*=0_ = [**w**^(0)^_*s*_]^*S*−1^_*s*=0_ = [**g**^(0)^_*s*_]^*S*−1^_*s*=0_ = [**λ**^(0)^_**1***l*_]^*L*−1^_*l*=0_ = [**λ**^(0)^_**2***a*_]^*S*−1^_*a*=0_ = **λ**^(0)^_**3**_ = **λ**^(0)^_**4**_ = **λ**^(0)^_**5**_ = **0**, where **0** is an *m* × *n* zero matrix.

In each iteration, we first solve the seven subproblems in the listed order: ‘[**r**_*l*_]^*L*−1^_*l*=0_-problem’, ‘[**w**_*s*_]^*S*−1^_*s*=0_-problem’, ‘[**g**_*s*_]^*S*−1^_*s*=0_-problem’, ‘**v**-problem’, ‘**u**-problem’, ‘**e**-problem’, ‘***ϵ***-problem’, and then we update the five Lagrange multipliers, namely [**λ**_**1l**_]^*L*−1^_*l*=0_, [**λ**_**2a**_]^*S*−1^_*a*=0_, **λ_3_**, **λ_4_**, **λ_5_** (see algorithm 1).

In this section, we only present the solution of the ‘**e**-problem’ and ‘***ϵ***-problem’ as well as the updated Lagrange multiplier **λ_5_**. We refer readers to [35] for a detailed explanation of the other subproblems and the other Lagrange multipliers.

*The **e**-problem*: Fix **u**, **v**, ***ϵ***, [**r**_*l*_]^*L*−1^_*l*=0_, [**w**_*s*_]^*S*−1^_*s*=0_, [**g**_*s*_]^*S*−1^_*s*=0_ and

3.3 $$\min_{e\in X}\left\{G^{*}\left(\frac{e}{\nu }\right)+\frac{\beta_{5}}{2}{∥e-\left(\chi_{D}^{c}\cdot^{\times }\epsilon -\frac{\lambda_{5}}{\beta_{5}}\right)∥}_{\ell_{2}}^{2}\right\}.$$

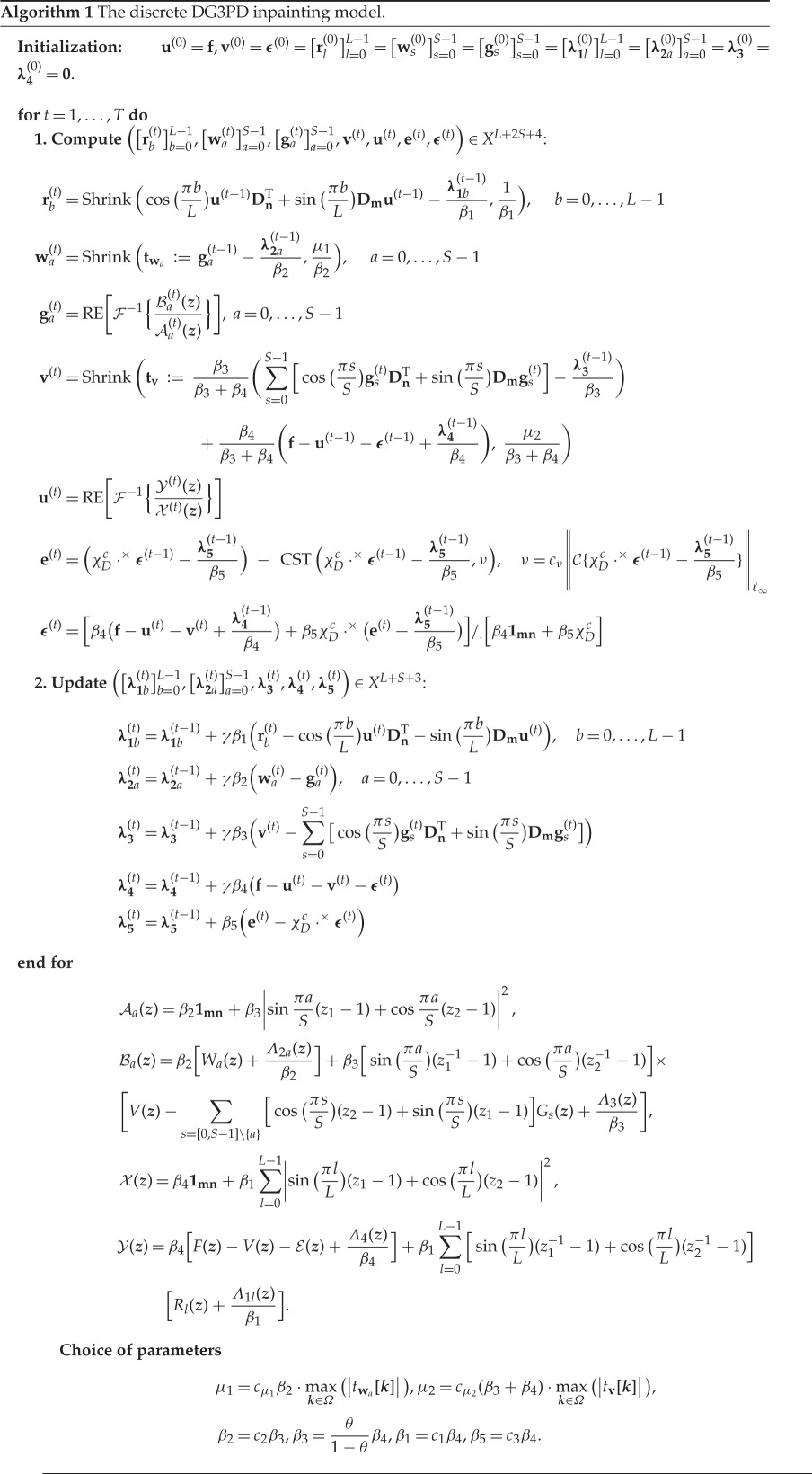


The solution of (3.3) is defined as a projection operator on a convex set *A*(*ν*), i.e. $P_{A(\nu )}(\chi_{D}^{c}\cdot^{\times }\epsilon -\lambda_{5}/\beta_{5})$:

3.4 $$e^{*}=\left(\chi_{D}^{c}\cdot^{\times }\epsilon -\frac{\lambda_{5}}{\beta_{5}}\right)-\mathrm{CST}\left(\chi_{D}^{c}\cdot^{\times }\epsilon -\frac{\lambda_{5}}{\beta_{5}},\nu \right).$$

*The **ϵ**-problem: Fix **u**, **v**, **e***, [**r**_*l*_]^*L*−1^_*l*=0_, [**w**_*s*_]^*S*−1^_*s*=0_, [**g**_*s*_]^*S*−1^_*s*=0_ and

3.5 $$\min_{\epsilon \in X}\left\{\frac{\beta_{4}}{2}{∥f-u-v-\epsilon +\frac{\lambda_{4}}{\beta_{4}}∥}_{\ell_{2}}^{2}+\frac{\beta_{5}}{2}{∥e-\chi_{D}^{c}\cdot^{\times }\epsilon +\frac{\lambda_{5}}{\beta_{5}}∥}_{\ell_{2}}^{2}\right\}.$$

The solution of (3.5) with the point-wise division operator /_._ is

3.6 $$\epsilon^{*}={[\beta_{4}\left(f-u-v+\frac{\lambda_{4}}{\beta_{4}}\right)+\beta_{5}\chi_{D}^{c}\cdot^{\times }\left(e+\frac{\lambda_{5}}{\beta_{5}}\right)]/}_{.}[\beta_{4}1_{mn}+\beta_{5}\chi_{D}^{c}]$$

and **1**_**mn**_ is an *m* × *n* matrix of ones.

*Updated Lagrange multiplier* **λ_5_**∈*X*:

$$\lambda_{5}=\lambda_{5}+\beta_{5}(e-\chi_{D}^{c}\cdot^{\times }\epsilon ).$$

Choice of parameters

Owing to an adaptability to specific images and a balance between the smoothing terms and updated terms for the solution of the above subproblems, the selection of parameters (*μ*_1_, *μ*_2_, *β*_1_, *β*_2_, *β*_3_) is described in [35] and *β*_5_ is defined as

$$\beta_{5}=c_{3}\beta_{4}.$$

**Figure 2.**
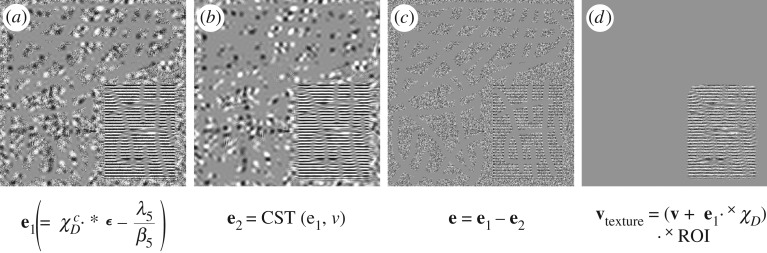
The ‘cancelling effect’ is caused by the noise measurement ${∥C\{\chi_{D}^{c}\cdot^{\times }\epsilon \}∥}_{\ell_{\infty }}$.

The term ${∥C\{\chi_{D}^{c}\cdot^{\times }\epsilon \}∥}_{\ell_{\infty }}$ in (2.1) serves as a good measurement for the amount of noise due to the substraction in equation (3.4) [35,36,53] (owing to the multi-scale and multi-direction properties of the curvelet transform and its duality). However, because of the indicator function *χ*^*c*^_*D*_ in the noise measurement ${∥C\{\chi_{D}^{c}\cdot^{\times }\epsilon \}∥}_{\ell_{\infty }}$, the interpolated texture by DG3PD inpainting is almost in the residual; see figure 2*a*, and its smoothed version by curvelet shrinkage (figure 2*b*). Because of the substraction operator in equation (3.4), these interpolated textures are cancelled out in **e** (figure 2*c*). Figure 2*d* illustrates the estimated texture before the substraction in equation (3.4) as

$$v_{\mathrm{texture}}=(v+e_{1}\cdot^{\times }\chi_{D})\cdot^{\times }\mathrm{ROI},$$

where ROI is the region of interest obtained by the morphological operator for the texture image in [37].

**Figure 3.**
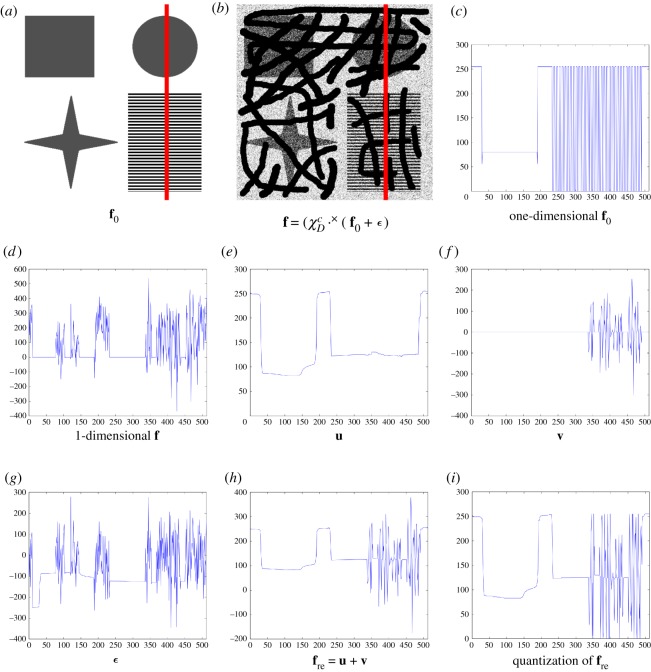
Interpolation for a one-dimensional signal after DG3PD inpainting (without texture inpainting): (*c*) a one-dimensional signal extracted along the red line in the original image (*a*). (*d*) The signal in (*c*) corrupted by Gaussian noise and missing regions *D*; see (*b*). Because of the directional TV norm, the DG3PD inpainting produces ‘sharp’ edges without a ‘staircase’ effect in a cartoon **u** (*e*) and the mean values of **u** remain almost unchanged. Owing to noise and the shrinkage soft-thresholding operator by ∥**v**∥_ℓ_1__ in (2.1), texture **v** is reconstructed with a ‘sharp’ transition on areas where texture presents in the original signal (*c*). However, due to ‘heavy’ noise ***ϵ*** on the known domains in (*e*), the DG3PD fails to reconstruct a ‘full’ texture. In general, this is a challenging problem to recover an oscillating (or weak) signal corrupted by heavy noise. (*h*) A reconstructed image (without the inpainting texture step) and (*i*) a quantized signal, i.e. truncated to [0, 255] and quantized.

In order to analyse the effects of the proposed model in terms of interpolation (for **u**) and decomposition, we consider a one-dimensional signal (which is extracted along the red line in figure 3*a*–*d*). By the DG3PD inpainting, the mean values of the cartoon **u** in (figure 3*e*) remain almost unchanged. The homogeneous regions have ‘sharp’ edges and a ‘staircase’ effect does not occur (owing to the directional TV). Texture **v** in (figure 3*f*) is extracted in areas which contain a repeated pattern (figure 3*b*). Moreover, small-scale objects, e.g. noise, are removed from **v**.

However, the term ${∥C\{\chi_{D}^{c}\cdot^{\times }\epsilon \}∥}_{\ell_{\infty }}$ causes a cancelling effect which removes the interpolated texture in an unknown area during the decomposition process. Therefore, texture inpainting and denoising are tackled separately, as described in the next section, by a generalized version of the dictionary learning for texture synthesis [30].

## 4. Texture inpainting and denoising

The proposed method for reconstructing the texture component combines the following ideas in five subsequent steps:

— texture extraction by DG3PD [35],
— morphological processing [36,37],
— texture inpainting inspired by the work of Efros & Leung [30] or [54],
— texture denoising by NL means [13],
— image synthesis by summation with the cartoon component **u**.

**Figure 4.**
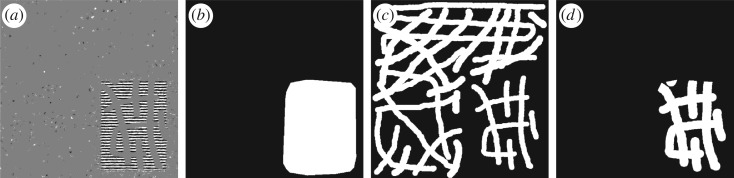
All coefficients of the obtained texture component **v** equal to zero are visualized by grey pixels in (*a*); positive coefficients are indicated by white pixels and negative coefficients by black pixels. (*b*) The resulting segmentation. The mask of missing pixels *D* shown in (*c*) is dilated and then intersected with (*b*), leading to the (white) pixels which are to be inpainted with texture in (*d*).

The DG3PD model enforces sparsity and smoothness on the obtained texture component **v**. Sparsity means that the vast majority of coefficients in **v** are equal to zero (the percentage of zero coefficients depends on how much texture a specific image contains). We make good use of this property to answer the following question: which pixels of those that are missing shall be inpainted with texture? First, the texture component **v** is segmented into zero (grey pixels in figure 4*a*) and non-zero coefficients (black and white pixels in figure 4*a*). Morphological processing (as described in [36,37] for fingerprint segmentation) obtains texture regions (figure 4*b*). The mask of missing pixels *D* shown in figure 4*c* is dilated (we used a circular structure element with a radius of 5 pixels to avoid border effects on the margin between existing and missing pixels) and then it is intersected with texture region segmentation to obtain the mask *I* shown in figure 4*d*, which shows the pixels to be inpainted with texture.

The proposed texture inpainting proceeds in two phases. First, we build a dictionary of texture patches and, second, we inpaint all pixels of mask *I* in a specific order. For the dictionary, we select all patches of size *s* × *s* pixels (we used *s* = 15 in our experiments) from texture component **v** which meet the following two criteria: (i) at least *p*_1_ per cent of the pixels are known (also the central pixel of the patch has to exist) and (ii) at least *p*_2_ per cent of the coefficients are non-zero (we used *p*_1_ = 70% and *p*_2_ = 60% in our experiments). The first criterion excludes patches which contain too many missing (unknown) pixels and the second criterion excludes patches without texture from the dictionary. Next, we iterate over all pixels of mask *I* and consider the pixel to be inpainted as the central pixel of an image patch with size *s* × *s*. We count the number of known pixels inside the patch and compute the percentage of known pixels. Pixels are inpainted in the following order. We start with a threshold percentage *t* = 90%; this is decreased in steps of 5% per iteration and, in each iteration, we inpaint all pixels of mask *I* for which at least *t* per cent are known. The rationale behind this ordering is that the more pixels are known (or already inpainted) in the neighbourhood around a missing pixel, the better it can inpainted, because this additional information improves the chances of finding a good match in the texture dictionary. A third constraint ensures that the overlap of known pixels between a dictionary patch and the patch around the missing pixels contains at least *p*_3_ per cent of known pixels (we used *p*_3_ = 30% in our experiments). Inpainting a pixel means that we find the best fitting patch in our texture dictionary and set the pixel value to the central pixel of that patch. Best fitting is defined as the minimum sum of squared differences per pixel, divided by the number of pixels which overlap.

**Figure 5.**
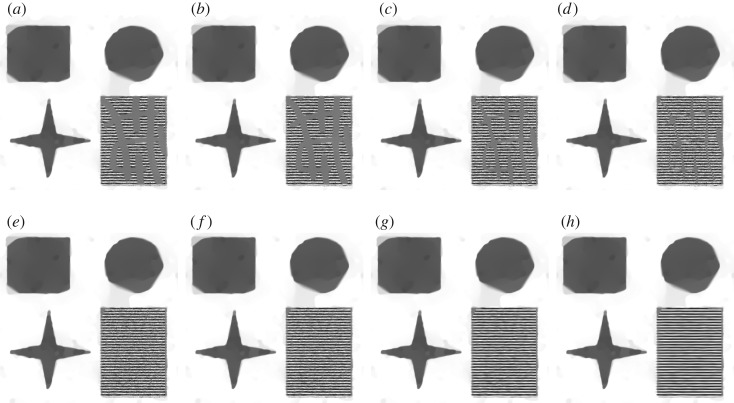
Images (*a*)–(*e*) visualize inpainting progress (added to the cartoon component) from 0% to 100% in steps of 25%. Images (*f*)–(*h*) display the denoising results after one, five and 20 iterations, respectively.

After all missing texture pixels have been inpainted, the texture region is denoised by *n* iterations of NL means. In each iteration, we first construct a dictionary considering all patches in the texture region with known and previously inpainted pixels. Next, for each pixel to be denoised, we find the top *k* fitting patches in the dictionary using the same distance function (sum of squared pixel-wise differences) and set the denoised pixel to the average value of the top *k* central pixel values. In our experiments, we used *k* = 5 and we have observed that, after about *n* = 10 iterations, the image has reached a steady state. See figure 5 for a visualization of the status at several intermediate steps during the inpainting and denoising process.

Finally, the full image is reconstructed by summation of the inpainted cartoon component **u** with the inpainted and denoised texture component **v** (figure 10).

## 5. Comparison of DG3PD with further inpainting and denoising methods

**Figure 6.**
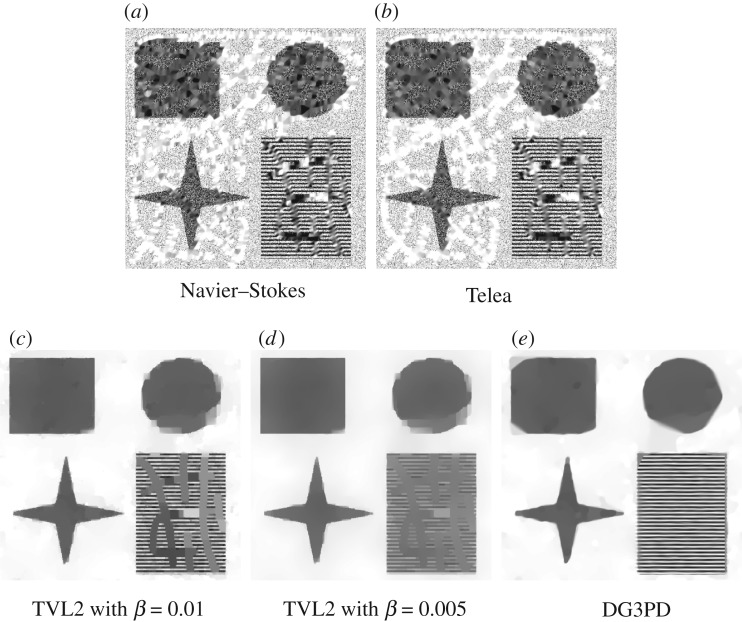
Comparison of inpainting results obtained by (*a*) Navier–Stokes [49], (*b*) Telea [48], (*c*, *d*) TVL2 and (*e*) DG3PD.

**Figure 7.**
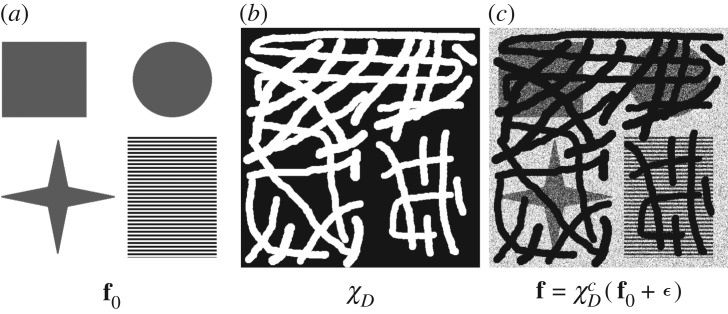
An ideal image (*a*) with cartoon and texture components. Missing pixels (*b*) are shown in white. Image (*c*) combines missing pixels and noise. $\epsilon [k]∼N(0,\sigma^{2}=100^{2}),k\in \Omega$.

Figure 6 shows inpainting results for the challenging problem under consideration (figure 7*c*) obtained by well-known inpainting methods from the literature: an approach based on Navier–Stokes equations from fluid dynamics which was proposed by Bertalmio *et al.* [49], an inpainting method suggested by Telea [48] and a well-known TVL2 inpainting approach with its synthesis image shown in figure 6*b*,*c*.

The image shown in figure 7*c* has the three properties discussed in §1:

(i) a large percentage of pixels in **f** are missing and shall be inpainted,
(ii) the known pixels in **f** are corrupted by noise,
(iii) **f** contains both cartoon and texture elements.

Furthermore, a noise-free, ideal version **f**_0_ depicted in figure 7*a* is available which serves as the ground truth for evaluating the quality of inpainted and denoised images by different methods. The availability of a noise-free image is an advantage for comparing and evaluating different inpainting and denoising methods. By contrast, images taken by a digital camera contain a certain amount of noise and, for them, an ideal version is not available, see, for example, the Barbara image we used in previous comparisons [35]. The choice of the ideal image (shown in figure 7) enables us to clearly see and understand the advantages and limitations of the compared methods (figure 6).

Owing to the fidelity term, the *L*_2_-norm, in the TVL2 inpainting model, noise is reduced and the homogeneous areas are well interpolated. However, the method is known to cause a ‘staircase’ effect on the homogeneous regions and texture, while small-scale objects tend to be eliminated. If the parameter *β* is chosen smaller (e.g. *β*_5_ = 0.005), the resulting inpainted image is smoother, i.e. the ‘staircase’ effect is reduced, but also more texture parts are removed.

DG3PD inpainting produces a smooth result without the staircase effect. In comparison with the other approaches, it is the only method which can reconstruct the texture regions in a satisfactory way.

In order to evaluate the proposed model in terms of image denoising, we compared it with BM3D in [50]. This NL-mean filter enhances the sparse representation of an image in a transform domain by grouping similar two-dimensional image blocks into three-dimensional ‘groups’. Collaborative filtering is applied for these three-dimensional groups by using three successive steps: three-dimensional transformation of a group, shrinkage of the transform spectrum, and inverse three-dimensional transformation. Finally, aggregation (i.e. an averaging procedure) is applied for different estimates of each pixel due to several overlapped blocks.

BM3D is a state-of-the-art method for image denoising. However, it is not designed for inpainting and, in order to be able to run the BM3D code available at http://www.cs.tut.fi/foi/GCF-BM3D/, we have to provide input values for the missing pixels; therefore, we

**Figure 8.**
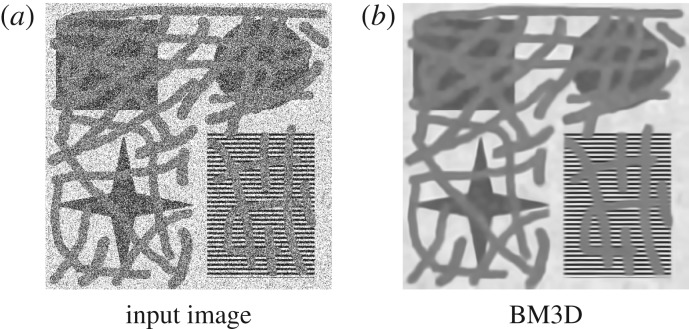
A performance of BM3D for image denoising. An input image (*a*) is obtained by setting the pixels in the missing regions of figure 7*c* to uniformly random values in [0, 255]. Image (*b*) is an output image of (*a*) by BM3D.

— set missing pixels in a corrupted image in figure 7*c* as uniform random values in [0, 255] (figure 8*a*),
— let standard deviation of noise in known regions *σ* = 100, and
— linearly rescale an input image to [0, 1].

In the end, we realized that, while BM3D can separate noise reasonably well from a corrupted image in figure 8*a*, the resultant image illustrated in figure 8*b* does not have a good performance. In particular,

— from an inpainting aspect, BM3D cannot interpolate mixing areas on homogeneous or textured regions and
— from a denoising aspect, reconstructed areas by BM3D are blurred without sharp edges and with a loss of contrast as well as with several artefacts on homogeneous regions.

## 6. From the Bayesian framework to the DG3PD inpainting model

Following [55], in this section, we describe the DG3PD inpainting model from the perspective of a Bayesian framework through a maximum *a posteriori* (MAP) estimator. We assume that **u** and **v** are independent random variables (r.v.).

— *Prior of cartoon image **u***: A cartoon **u** = [*u*[***k***]]_*k*∈*Ω*_ consists of an element pixel *u*[***k***] which is considered as an independent r.v. with a prior *p*_*U*_*k*__.
  Given ***k***∈*Ω*, denote *r*[***k***] = ∇^+^_*L*_*u*[***k***] = [*r*_*l*_[***k***]]^*L*−1^_*l*=0_. To describe the distribution of a r.v. *U*_*k*_, we firstly define the distribution of an *L*-variate r.v. ***R***_*k*_ = [*R*_0,*k*_, …, *R*_*L*−1,*k*_]. We assume that r.v. ***R***_*k*_ has a multi-variate Laplace distribution which is a part of multi-variate power exponential distribution [56–58], i.e. $R_{k}∼{\mathrm{PE}}_{L}(0,\Sigma ,\frac{1}{2})={\mathrm{Laplace}}_{L}(0,\Sigma )$ with $\Sigma =\left(\begin{array}{ccc} \gamma^{2} & \ldots & 0\\ \vdots & \ddots & \vdots \\ 0 & \ldots & \gamma^{2} \end{array}\right)\in R_{+}^{L\times L}\setminus \{0\}$ and *Γ*( · ) function:
  $$\begin{array}{l} p_{R_{k}}\left(r[k]|0,\Sigma ,\frac{1}{2}\right)\\ \;=\frac{\Gamma (L/2)}{\pi^{L/2}\Gamma (L)2^{1+L}\gamma^{L}}\exp\left\{-\frac{1}{2}{\left({\left(\begin{array}{c} r_{0}[k]\\ \vdots \\ r_{L-1}[k] \end{array}\right)}^{\text{T}}\frac{1}{\gamma^{2}}\left(\begin{array}{ccc} 1 & \ldots & 0\\ \vdots & \ddots & \vdots \\ 0 & \ldots & 1 \end{array}\right)\left(\begin{array}{c} r_{0}[k]\\ \vdots \\ r_{L-1}[k] \end{array}\right)\right)}^{1/2}\right\}\\ \;=\frac{\Gamma (L/2)}{\pi^{L/2}\Gamma (L)2^{1+L}\gamma^{L}}\underset{:=|r[k]|=|\nabla_{L}^{+}u[k]|}{\underset{︸}{{\left[\sum_{l=0}^{L-1}|r_{l}[k]{|}^{2}\right]}^{1/2}}}. \end{array}$$
  Thus, the distribution of a r.v. *U*_*k*_ is a multi-dimensional Laplace distribution with operator ∇^+^_*L*_:
  $$p_{U_{k}}(u[k])=\frac{\Gamma (L/2)}{\pi^{L/2}\Gamma (L)2^{1+L}\gamma^{L}}\exp\left\{-\frac{1}{2\gamma }\left|\nabla_{L}^{+}u[k]\right|\right\}.$$
  The joint probability density function (p.d.f.) of a prior **u** is
  $$p_{U}(\text{u})=\prod_{k\in \Omega }p_{U_{k}}(u[k])={\left[\frac{\Gamma (L/2)}{\pi^{L/2}\Gamma (L)2^{1+L}\gamma^{L}}\right]}^{\left|\Omega \right|}e^{-(1/2\gamma )}{∥\nabla_{L}^{+}\text{u}∥}_{\ell_{1}}.$$
  We choose $\gamma =\frac{1}{2}$. The potential function of **u** in matrix form is
  $$\Phi_{U}(\text{u})=-\log p_{U}(\text{u})={∥\nabla_{L}^{+}\text{u}∥}_{\ell_{1}}+|\Omega |\log\frac{2\pi^{L/2}\Gamma (L)}{\Gamma (L/2)}.$$
  Note that the original **u** is not a Laplace distribution, but a transform of **u** under an operator ∇^+^_*L*_ has an independent multi-dimensional Laplace distribution.
— *Prior of texture image **v***: Under a transform operator T, the texture image **v** is decomposed in different orientations. Operator T is suitably chosen to capture the texture components in the original image **f**. As for the definition of a discrete multi-dimensional G-norm [35], the transform T (in a direction *s*, *s* = 0, …, *S* − 1) is defined by its inversion (note that $TT^{-1}=\mathrm{Id}$):
  $$T\{v\}={\left[\underset{:=g_{s}}{\underbrace{T_{s}\{v\}}}\right]}_{s=0}^{S-1}=g\;\Leftrightarrow \;v=T^{-1}\{{\left[g_{s}\right]}_{s=0}^{S-1}\}={\mathrm{div}}_{S}^{+}g.$$
  We assume that a texture **v** is sparse in a transform domain T and sparse in the spatial domain (non-zero coefficients of **v** are only due to texture). To satisfy these conditions, we assume that a r.v. *V*_*k*_ has a mixture of a Laplace distribution in a spatial domain with a p.d.f. *p*_*V*_*k*_,2_(*v*[***k***]) (w.r.t. sparse in the spatial domain) and a multi-variate Laplace distribution (in an operator T) with a p.d.f. *p*_*V*_*k*_,1_(*v*[***k***]) (w.r.t. sparse in the transform domain T). Thus, we have a p.d.f. of r.v. *V*_*k*_ as follows:
  6.1 $$p_{V_{k}}(v[k])=p_{V_{1k}}(v[k])\cdot p_{V_{2k}}(v[k]).$$
  Since *V*_2*k*_∼Laplace(0, *γ*^2^_1_), we have
  6.2 $$p_{V_{2k}}(v[k])=\frac{1}{4\gamma_{2}}\exp\left\{-\frac{1}{2\gamma_{2}}\left|v[k]\right|\right\}.$$
  To define the distribution of a r.v. *V*_1*k*_, we define the distribution of the *S*-dimension r.v. ***G***_*k*_ = [*G*_0,*k*_, …, *G*_*S*−1,*k*_]. Similar to ***U***_*k*_, we assume that r.v. ***G***_*k*_ has a multi-variate Laplace distribution, i.e. ***G***_*k*_∼Laplace_*S*_(0, *Σ*) with $\Sigma =\left(\begin{array}{ccc} \gamma_{1}^{2} & \ldots & 0\\ \vdots & \ddots & \vdots \\ 0 & \ldots & \gamma_{1}^{2} \end{array}\right)\in R_{+}^{S\times S}\setminus \{0\}$. It is easy to see that (with g[k]=T{v}[k],k∈Ω)
  $$p_{{\text{G}}_{\text{k}}}(g[k])=\frac{\Gamma (S/2)}{\pi^{S/2}\Gamma (S)2^{1+S}\gamma_{1}^{S}}\exp\left\{-\frac{1}{2\gamma_{1}}\left|g[k]\right|\right\}$$
  or
  6.3 $$p_{V_{1k}}(v[k])=\frac{\Gamma (S/2)}{\pi^{S/2}\Gamma (S)2^{1+S}\gamma_{1}^{S}}\exp\left\{-\frac{1}{2\gamma_{1}}\left|T\{\text{v}\}[k]\right|\right\}.$$
  Putting (6.2) and (6.3) into (6.1), we have the p.d.f. of r.v. *V*_*k*_ as
  $$p_{V_{k}}(v[k])=\frac{\Gamma (S/2)}{\pi^{S/2}\Gamma (S)2^{3+S}\gamma_{1}^{S}\gamma_{2}}\exp\left\{-\frac{1}{2\gamma_{1}}\left|T\{\text{v}\}[k]\right|-\frac{1}{2\gamma_{2}}\left|v[k]\right|\right\}.$$
  The joint p.d.f. of a texture image **v** = [*v*[***k***]]_***k***∈*Ω*_ is
  $$p_{V}(\text{v})=\prod_{k\in \Omega }p_{V_{k}}(v[k])={\left[\frac{\Gamma (S/2)}{\pi^{S/2}\Gamma (S)2^{3+S}\gamma_{1}^{S}\gamma_{2}}\right]}^{\left|\Omega \right|}\exp\left\{-\frac{1}{2\gamma_{1}}{∥T\{\text{v}\}∥}_{\ell_{1}}-\frac{1}{2\gamma_{2}}{∥\text{v}∥}_{\ell_{1}}\right\},$$
  and we choose *μ*_2_ = 1/2*γ*_2_ and *μ*_1_ = 1/2*γ*_1_. The potential function of **v** with an anisotropic version $\left(∥T\{v\}{∥}_{\ell_{1}}=\sum_{s=0}^{S-1}∥T_{s}\{v\}{∥}_{\ell_{1}}=\sum_{s=0}^{S-1}∥g_{s}{∥}_{\ell_{1}}\right)$ in matrix form is defined as
  $$\Phi_{V}(\text{v})=\mu_{1}\sum_{s=0}^{S-1}{∥{\text{g}}_{\text{s}}∥}_{\ell_{1}}+\mu_{2}∥\text{v}{∥}_{\ell_{1}}+|\Omega |\log\frac{4\pi^{S/2}\Gamma (S)}{\Gamma (S/2)\mu_{1}^{S}\mu_{2}}.$$
— *Likelihood (or the joint p.d.f. of *p*_*F*|*U*,*V*_ (**f**|**u**, **v**))*: If we assume that the residual ***ϵ*** in an image **f** has a power exponential distribution [56,57], i.e. *E*_*k*_∼PE(*μ*, *σ*^2^, *ξ*), e.g. Gaussian (*ξ* = 1) or Laplacian $\left(\xi =\frac{1}{2}\right)$, its density function at ***k***∈*Ω* is
  $$p_{E_{k}}(\epsilon [k]|\mu )=\frac{1}{\Gamma (1+1/2\xi )2^{1+1/2\xi }\sigma }\exp\left\{-\frac{1}{2}\frac{{\left|\epsilon [k]-\mu \right|}^{2\xi }}{\sigma^{2\xi }}\right\}.$$
  Owing to the inpainting problem with a missing region *D*, the likelihood is defined on a known domain *Ω*\*D* as
  $$\begin{array}{c} p_{F|U,V}(f|u,v)=\prod_{k\in \Omega }p_{F_{k}|U_{k},V_{k}}(f[k]|u[k],v[k])\;\\ \;=\frac{1}{{\left(\Gamma (1+1/2\xi )2^{1+1/2\xi }\sigma \right)}^{|\Omega |}}\exp\left\{-\frac{1}{2\sigma^{2\xi }}\underset{:{∥=\chi_{D}^{c}\cdot^{\times }(f-u-v)∥}_{2\xi }^{2\xi }}{\underbrace{\sum_{k\in \Omega }{\left|\chi_{D}^{c}[k](f[k]-u[k]-v[k])\right|}^{2\xi }}}\right\}.\; \end{array}$$
— *A posteriori*: Since **u** and **v** are independent of each other, *a posteriori* is written as
  $$p_{U,V|F}(u,v|f)=\frac{p_{F|U,V}(f|u,v)p_{U}(u)p_{V}(v)}{p_{F}(f)}\propto p_{F|U,V}(f|u,v)p_{U}(u)p_{V}(v).$$

Let ***ϵ*** = **f** − **u** − **v**, and the MAP estimator, i.e. $\max_{(u,v)\in X^{2}}p_{U,V|F}(u,v|f)$, is defined as

$$\begin{array}{rl} & \min_{(u,v)\in X^{2}}\left\{\underset{=\Phi_{U}(u)}{\underbrace{-\log p_{U}(u)}}\;\underset{=\Phi_{V}(v)}{\underbrace{-\log p_{V}(v)}}-\log p_{U,V|F}(u,v|f)\right\}\\ & \;=\min_{(u,v)\in X^{2}}\{{∥\nabla_{L}^{+}u∥}_{\ell_{1}}+\mu_{1}\sum_{s=0}^{S-1}{∥g_{s}∥}_{\ell_{1}}+\mu_{2}∥v{∥}_{\ell_{1}}+\frac{1}{2\sigma^{2\xi }}{∥\chi_{D}^{c}\cdot^{\times }\epsilon ∥}_{2\xi }^{2\xi },\\ & \;s.t.\;v=\sum_{s=0}^{S-1}\left[\cos\left(\frac{\pi s}{S}\right)g_{s}D_{n}^{T}+\sin\left(\frac{\pi s}{S}\right)D_{m}g_{s}\right],f=u+v+\epsilon \}. \end{array}$$

However, in practice, we do not know which types of noise are observed in a signal. Instead of the ℓ_2*ξ*_ norm of the residual ***ϵ*** on a known domain *Ω*\*D* to characterize the properties of noise, we control the smoothness of the solution by the maximum of the curvelet coefficient of ***ϵ*** on a domain *Ω*\*D* by a constant *ν*, i.e. ${∥C\{\chi_{D}^{c}\cdot^{\times }\epsilon \}∥}_{\ell_{\infty }}\leq \nu$.

In [59], if noise in image **f** is Gaussian, as in extreme value theory, the r.v. ${∥C\{\chi_{D}^{c}\cdot^{\times }\epsilon \}∥}_{\ell_{\infty }}$ has a Gumbel distribution. Thus, there is a condition to choose *ν*. However, in practice, if noise cannot be identified, *ν* is chosen by *α*-quantile. Note that the condition ${∥C\{\chi_{D}^{c}\cdot^{\times }\epsilon \}∥}_{\ell_{\infty }}\leq \nu$ is similar to the Dantzig selector [60].

**Figure 9.**
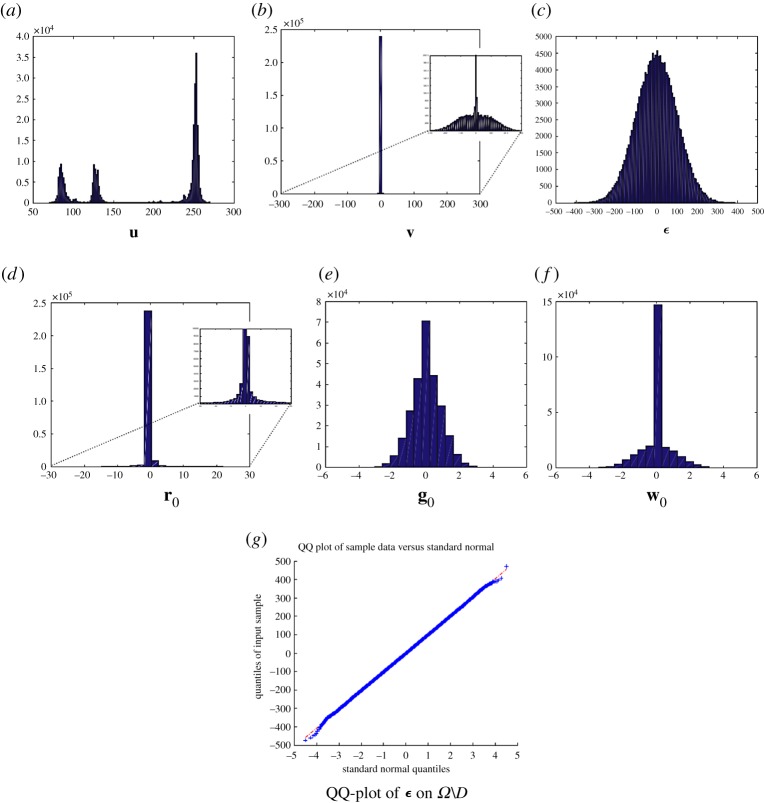
The empirical density functions of the solution in (3.2) by an alternating direction method of multipliers: (*a*)–(*g*) are the empirical density functions of **u**, **v**, ***ϵ***, **r**_**0**_, **g**_**0**_, **w**_**0**_ and the QQ-plot of the residual ***ϵ***, respectively. We assume that the prior of ∇^+^_*L*_**u** has a multi-Laplacian distribution, i.e. a changed variable **r** = [**r**_*l*_]^*L*−1^_*l*=0_ = ∇^+^_*L*_**u** has a multi-Laplace distribution, or **r**_*l*_ has a univariate Laplace distribution. This effect causes the sparseness of **r**_*l*_ due to the shrinkage operator; see (*d*). The sparsity for ∇^+^_*L*_**u** causes the smoothness of the objects in cartoon **u** (*a*). The smooth and sparse texture **v** (due to the expansion of the whole range in intensity value of its non-zero coefficients caused by ∥**v**∥_ℓ_1__ in (2.1)) is illustrated in (*b*). Because of an additive white Gaussian noise in a simulation, the distribution of ***ϵ*** in *Ω*\*D* is approximately normal; see plot (*c*) and its QQ-plot (*g*). The reasons for the differences near the boundary in (*g*) may be as follows. (1) A simulation of a Gaussian noise, i.e. the tail of a Gaussian noise does not go to infinity in a simulation. (2) The remaining texture in ***ϵ*** (due to a selection of *ν*). Changing variable **w** = **g** causes a non-sparsity in **g** and a sparsity in **w** (which is shrunk from **g**); c.f. (*e*) and (*f*).

Figure 9 illustrates the empirical density functions of the solution of the minimization (3.2). The statistical properties of the solution can be characterized in the Bayesian framework with the priors, likelihood and posterior as mentioned above.

**Figure 10.**
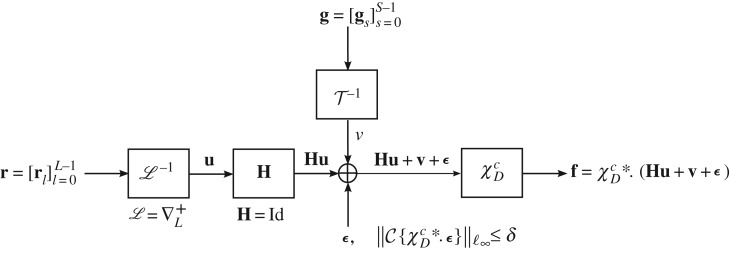
Overview of the discrete innovation model for the DG3PD-based image inpainting and denoising. In this study, a blurring operator **H** is an identity matrix. Note that the $L^{-1}$ does not exist, in general, but the reconstructed image is obtained by the function space of the inverse problem.

Note that the DG3PD inpainting model (2.1) can be described as an inverse problem in image restoration (e.g. image inpainting and denoising); see figure 10 for the discrete innovation model.

## 7. Relation between variational analysis and pyramid decomposition

In this section, we discuss connections and similarities between the proposed DG3PD inpainting model and existing work in the area of pyramid representations, multi-resolution analysis and scale-space representation. Historically, Gaussian pyramids and Laplacian pyramids have been developed for applications such as texture synthesis, image compression and denoising. Early works in this area include a paper by Burt & Adelson [42] applying Laplacian pyramids for image compression. Pyramidal decomposition schemes [42–44] can be grouped into two categories. Their main property is either lowpass or bandpass filtering. Closely related are transforms from multi-resolution analysis, including wavelet, curvelet [47,53], contourlet [61] and steerable wavelet [62]. A difference in the proposed DG3PD inpainting model is that its basis elements for obtaining the decomposition are constructed by discrete differential operators and the decomposition is solved in a nonlinear way which adapts to each image (enabled by update iterations, corresponding to the loop in figure 15). The discussion of commonalities and differences is made more precise and detailed in the rest of the section.

Let us denote $\overset{F}{⟷}$ as a discrete Fourier transform pair. Given *z*_1_ = *e*^*jω*_1_^ and *z*_2_ = *e*^*jω*_2_^ and the Dirac delta function *δ*( · ), the impulse responses of the discrete directional derivative operators (*l* = 0, …, *L* − 1) and their spectra are

— the forward operator:
  $$\begin{array}{rl} \partial_{l}^{+}\delta [k] & =\left[\cos\left(\frac{\pi l}{L}\right)\partial_{x}^{+}+\sin\left(\frac{\pi l}{L}\right)\partial_{y}^{+}\right]\delta [k]\\ & \overset{F}{⟷}\cos\left(\frac{\pi l}{L}\right)(z_{2}-1)+\sin\left(\frac{\pi l}{L}\right)(z_{1}-1), \end{array}$$
— the backward operator:
  $$\begin{array}{rl} \partial_{l}^{-}\delta [k] & =\left[\cos\left(\frac{\pi l}{L}\right)\partial_{x}^{-}+\sin\left(\frac{\pi l}{L}\right)\partial_{y}^{-}\right]\delta [k]\\ & \overset{F}{⟷}-\cos\left(\frac{\pi l}{L}\right)(z_{2}^{-1}-1)-\sin\left(\frac{\pi l}{L}\right)(z_{1}^{-1}-1). \end{array}$$

### 7.1. The ‘**u**-problem’

In order to describe the variational analysis for the **u**-problem from the point of view of filtering in the Fourier domain for a pyramid decomposition scheme, we define the discrete inverse Fourier transform of X(z) and Y(z) in the ‘**u**-problem’ in [35] as

$$\begin{array}{rl} X(z)\overset{F^{-1}}{⟷}X[k] & =\left[\beta_{4}-\beta_{1}\sum_{l=0}^{L-1}\partial_{l}^{-}\partial_{l}^{+}\right]\delta [k]\\ \;\mathrm{and}\;Y(z)\overset{F^{-1}}{⟷}Y[k] & =\beta_{4}\left(f[k]-v[k]-\epsilon [k]+\frac{\lambda_{4}[k]}{\beta_{4}}\right)\\ & \;-\beta_{1}\sum_{l=0}^{L-1}\partial_{l}^{-}\left\{\underset{:=\;r_{l}[k]}{\underbrace{\mathrm{Shrink}(\partial_{l}^{+}u[k]-\frac{\lambda_{1l}[k]}{\beta_{1}},\frac{1}{\beta_{1}})}}+\frac{\lambda_{1l}[k]}{\beta_{1}}\right\}. \end{array}$$

Note that the function X(z) satisfies

$$0<\beta_{4}\leq X(z)<+\infty ,\;\forall \omega \in {\left[-\pi ,\pi \right]}^{2}$$

and is similar to the autocorrelation function in [63], which follows the condition of the Riesz basis. We rewrite cartoon **u** in the Fourier and spatial domain in the scheme of the filter design as

7.1 $$\begin{array}{rl} U(z) & =X^{-1}(z)\cdot Y(z)=\Phi (z)\left(F(z)-V(z)-E(z)+\frac{\Lambda_{4}(z)}{\beta_{4}}\right)+\sum_{l=0}^{L-1}\tilde{\Psi }(z)\left[R_{l}(z)+\frac{\Lambda_{1\;l}(z)}{\beta_{1}}\right],\\ \overset{F^{-1}}{⟷}u[k] & =\underset{:=u_{\mathrm{update}}[k]}{\underbrace{\left(\phi *\left(f-v-\epsilon +\frac{\lambda_{4}}{\beta_{4}}\right)\right)[k]}}\\ & \;+\underset{:={\mathrm{FST}}_{\psi ,\tilde{\psi },c_{1}}(u,\lambda_{1l}/\beta_{1},1/\beta_{1}):=u_{\mathrm{smooth}}[k]\;\left(\mathrm{framesoftthresholding}\right)}{\underbrace{\sum_{l=0}^{L-1}\left({\tilde{\psi }}_{l}*\left[\mathrm{Shrink}\left(\psi_{l}*u-\frac{\lambda_{1\;l}}{\beta_{1}},\frac{1}{\beta_{1}}\right)+\frac{\lambda_{1l}}{\beta_{1}}\right]\right)[k]}}. \end{array}$$

Given *β*_1_ = *c*_1_*β*_4_, the spectra and impulse responses of the scaling and frame functions in (7.1) (*l* = 0, …, *L* − 1) are

$$\begin{array}{rl} \Phi (z) & =\beta_{4}X^{-1}(z)\overset{F}{⟷}\phi [k]={\left[1-c_{1}\sum_{l=0}^{L-1}\partial_{l}^{-}\partial_{l}^{+}\right]}^{-1}\delta [k],\\ {\tilde{\Psi }}_{l}(z) & =\beta_{1}X^{-1}(z)\left[(z_{1}^{-1}-1)\sin\frac{\pi l}{L}+(z_{2}^{-1}-1)\cos\frac{\pi l}{L}\right]\overset{F}{⟷}{\tilde{\psi }}_{l}[k]\\ & =-c_{1}{\left[1-c_{1}\sum_{l=0}^{L-1}\partial_{l}^{-}\partial_{l}^{+}\right]}^{-1}\partial_{l}^{-}\delta [k]\\ \;\mathrm{and}\;\Psi_{l}(z) & =(z_{1}-1)\sin\frac{\pi l}{L}+(z_{2}-1)\cos\frac{\pi l}{L}\overset{F}{⟷}\psi_{l}[k]=\partial_{l}^{+}\delta [k]. \end{array}$$

Equation (7.1) is somewhat similar to the projection operator in the pyramid decomposition scheme (see curvelet [47,53], contourlet [61], steerable wavelet [62], etc.) with:

— scaling function *φ*( · ) and its dual $\tilde{\varphi }(\cdot )$ with the interpolant $\phi =\varphi *\tilde{\varphi }$,
— frame function *ψ*_*l*_( · ) and its dual ${\tilde{\psi }}_{l}(\cdot )$,
— proximity operator for frame elements, i.e. Shrink( · , · ).

Note that the frame soft-thresholding FST( · , · , · ) in (7.1) consists of frame element *ψ*_*l*_, its dual ${\tilde{\psi }}_{l}$ and shrinkage operator Shrink( · , · ) together with the updated Lagrange multiplier. Although FST( · , · , · ) is similar to the wavelet/curvelet shrinkage operator, this operator is obtained from the calculus of variation.

**Figure 11.**
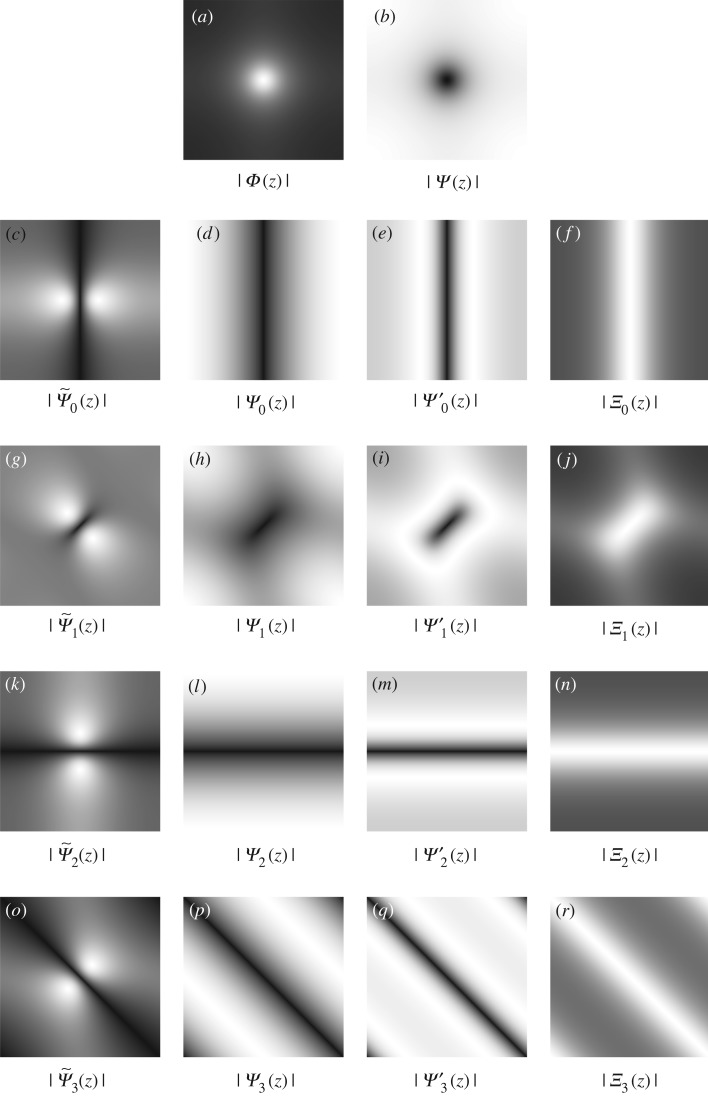
The spectra of frame functions in the ‘**u**-problem’ and the ‘**g**-problem’ with the parameters: *L* = *S* = 4, *β*_1_ = *β*_4_ = 0.04, *β*_2_ = *β*_3_ = 0.3 and $\Psi (z)=\sum_{l=0}^{L-1}{\tilde{\Psi }}_{l}(z)\Psi_{l}(z)$. The unity properties for these frame functions are $\Phi (z)+\sum_{l=0}^{L-1}{\tilde{\Psi }}_{l}(z)\Psi_{l}(z)=1$ and *Ξ*_*a*_(***z***) + *Ψ*_*a*_(***z***)*Ψ*′_*a*_(***z***) = 1, *a* = 0, …, *S* − 1.

**Figure 12.**
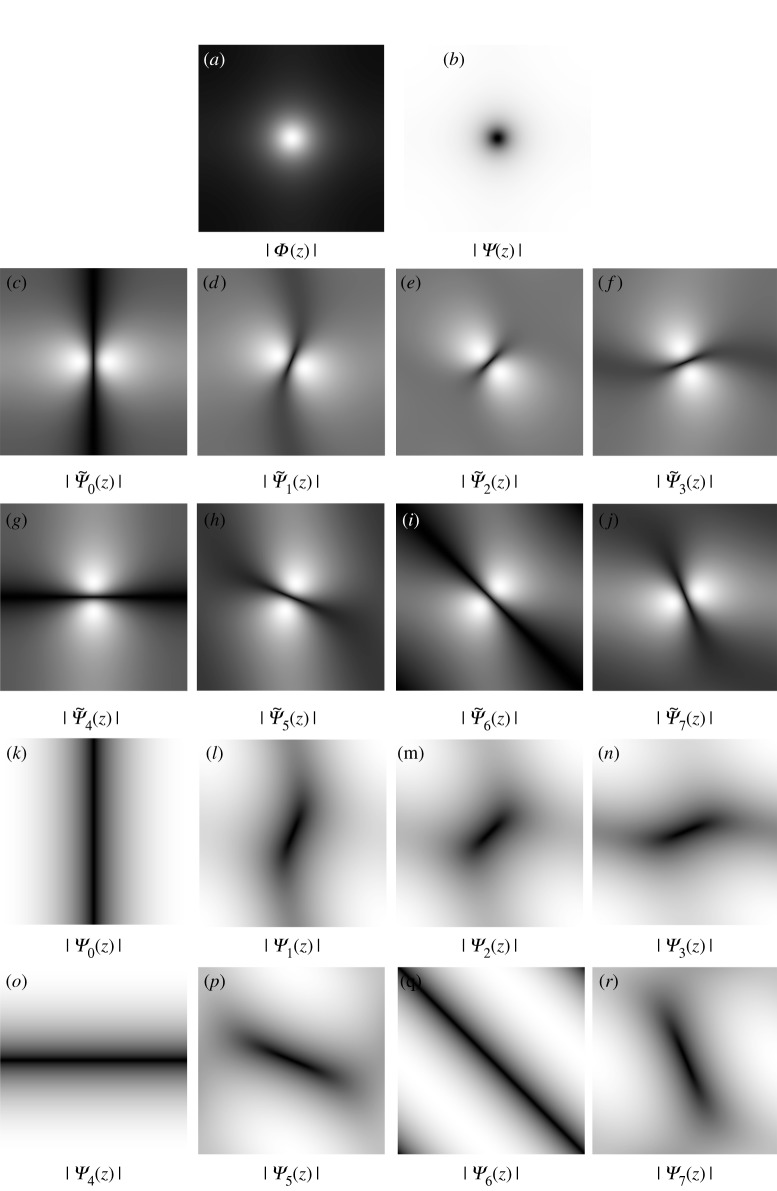
The spectrum of the frame functions in the **u** and **g** problems with the parameters *L* = *S* = 8, *β*_1_ = *β*_4_ = 0.04, *β*_2_ = *β*_3_ = 0.3 and $\Psi (z)=\sum_{l=0}^{L-1}{\tilde{\Psi }}_{l}(z)\Psi_{l}(z)$; see figure 13 for more directional frame functions of the **g** problem.

It is easy to obtain the unity property of frame elements $\left(\phi ,\psi_{l},{\tilde{\psi }}_{l}\right)$ in the Fourier domain as

$$\begin{array}{rl} & \Phi (z)+\sum_{l=0}^{L-1}{\tilde{\Psi }}_{l}(z)\Psi_{l}(z)=1,\\ & \Phi (e^{j0})=1\;\mathrm{and}\;{\tilde{\Psi }}_{l}(e^{j0})=\Psi_{l}(e^{j0})=0, \end{array}$$

which satisfies the condition of the perfect reconstruction in the scheme of the wavelet pyramid; see figure 11 for illustration with *L* = 4 directions. When the number of directions *L* increases, the bandwidth of the scaling function *Φ*(*z*) in the spectral domain reduces (figure 12). Owing to the effect of this lowpass filter, the cartoon **u** is smoother. These frame elements $\left(\phi ,\psi_{l},{\tilde{\psi }}_{l}\right)$, which consist of partial differential operators, can be considered as a kind of a wavelet-like operator [45]. However, the procedure to obtain these elements is from the calculus of variation.

**Figure 13.**
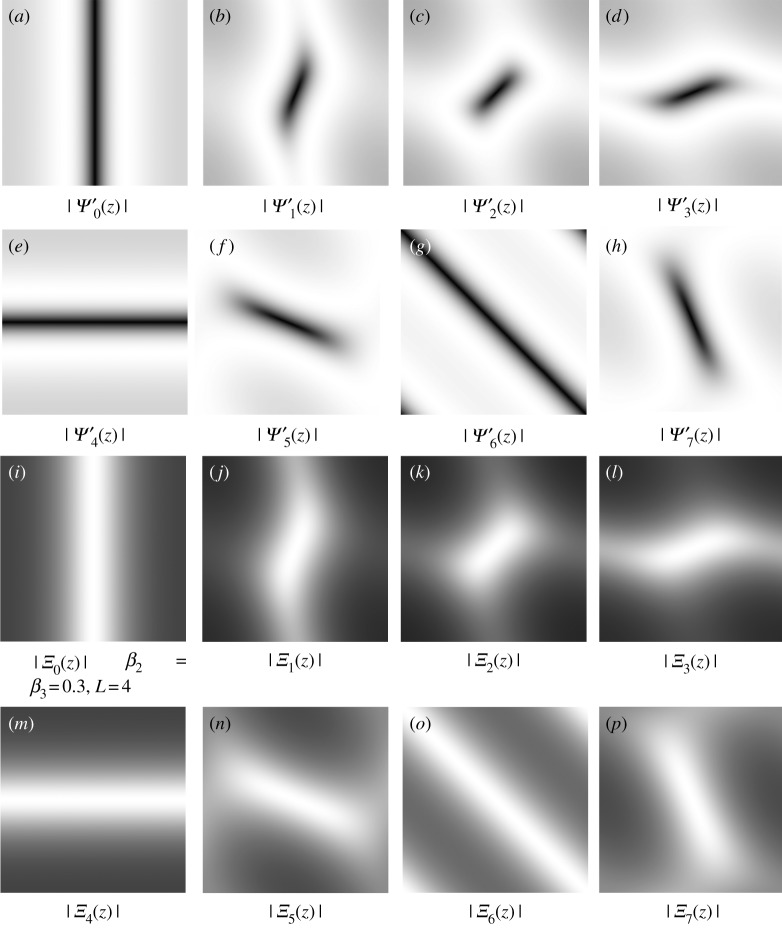
The spectrum of the frame functions in the **g**-problem with *S* = 8 directions.

### 7.2. The ‘[**g**_*s*_]^*S*−1^_*s*=0_-problem’

In analogy to the **u**-problem in the variational framework, we define the frame operators to extract the directional features of the texture **v** in the pyramid decomposition scheme. The discrete inverse Fourier transforms of A(z) and B(z) in the solution of the ‘**v**-problem’ [35] are defined as

$$\begin{array}{rl} A_{a}(z)\overset{F^{-1}}{⟷}A_{a}[k] & =[\beta_{2}-\beta_{3}\partial_{a}^{-}\partial_{a}^{+}]\delta [k]\\ \;\mathrm{and}\;B_{a}(z)\overset{F^{-1}}{⟷}B_{a}[k] & =\beta_{2}\left[\mathrm{Shrink}\left(g_{a}[k]-\frac{\lambda_{2a}[k]}{\beta_{2}},\frac{\mu_{1}}{\beta_{2}}\right)+\frac{\lambda_{2a}[k]}{\beta_{2}}\right]\\ & \;-\beta_{3}\partial_{a}^{-}\left\{\underset{:=\partial_{a}^{+}g_{a}[k]}{\underbrace{v[k]-\sum_{s=[0,S-1]\setminus \{a\}}\partial_{s}^{+}g_{s}[k]}}+\frac{\lambda_{3}[k]}{\beta_{3}}\right\}. \end{array}$$

Similar to X(z), the ‘autocorrelation’ function $A_{a}(z)$ is bounded as in the condition of the Riesz basis [63], i.e.

$$0<\beta_{2}\leq A_{a}(z)<+\infty ,\;\forall \omega \in {\left[-\pi ,\pi \right]}^{2}.$$

Texture **v** is rewritten in the Fourier and spatial domains with *a* = 0, …, *S* − 1, as

7.2 $$\begin{array}{rl} & G_{a}(z)=A_{a}^{-1}(z)\cdot B_{a}(z)=\Xi_{a}(z)\left[W_{a}(z)+\frac{\Lambda_{2a}(z)}{\beta_{2}}\right]+\Psi_{a}^{\prime }(z)\left[\Psi_{a}(z)G_{a}(z)+\frac{\Lambda_{3}(z)}{\beta_{3}}\right]\\ & \;\overset{F^{-1}}{⟷}\;g_{a}[k]=\left(\xi_{a}*\left[\underset{:=\mathrm{ST}(g_{a},\lambda_{2a}/\beta_{2},\mu_{1}/\beta_{2})}{\underbrace{\mathrm{Shrink}\left(g_{a}-\frac{\lambda_{2a}}{\beta_{2}},\frac{\mu_{1}}{\beta_{2}}\right)+\frac{\lambda_{2a}}{\beta_{2}}}}\right]\right)[k]+\left(\psi_{a}^{\prime }*\left[\psi_{a}*g_{a}+\frac{\lambda_{3}}{\beta_{3}}\right]\right)[k]. \end{array}$$

Given *β*_2_ = *c*_2_*β*_3_, the spectra and impulse responses of the frame functions in (7.2) are

$$\begin{array}{rl} \Xi_{a}(z) & =\beta_{2}A^{-1}(z)\overset{F^{-1}}{⟷}\xi_{a}[k]=c_{2}{\left(c_{2}-\partial_{a}^{-}\partial_{a}^{+}\right)}^{-1}\delta [k],\\ \Psi_{a}^{\prime }(z) & =\beta_{3}A^{-1}(z)\left[(z_{1}^{-1}-1)\sin\frac{\pi a}{S}+(z_{2}^{-1}-1)\cos\frac{\pi a}{S}\right]\overset{F^{-1}}{⟷}\psi_{a}^{\prime }[k]\\ & =-{\left(c_{2}-\partial_{a}^{-}\partial_{a}^{+}\right)}^{-1}\partial_{a}^{-}\delta [k]\\ \;\mathrm{and}\;\Psi_{a}(z) & =(z_{2}-1)\cos\frac{\pi a}{S}+(z_{1}-1)\sin\frac{\pi a}{S}\overset{F^{-1}}{⟷}\psi_{a}[k]=\partial_{a}^{+}\delta [k]. \end{array}$$

Similar to the **u**-problem, it is easy to obtain the unity property of frame elements (*ξ*, *ψ*_*a*_, *ψ*′_*a*_) with *a*∈[0, *S* − 1] in the Fourier domain:

$$\begin{array}{rl} & \Xi_{a}(z)+\Psi_{a}(z)\Psi_{a}^{\prime }(z)=1,\\ & \Xi_{a}(e^{j0})=1\;\mathrm{and}\;\Psi_{a}(e^{j0})=\Psi_{a}^{\prime }(e^{j0})=0, \end{array}$$

which satisfies the condition of the perfect reconstruction; see figure 11 with *L* = 4 directions and figures 12 and 13 with *L* = 8 directions.

### 7.3. The ‘**v**-problem’

The solution to the **v**-problem is rewritten as

$$v^{*}=\text{Shrink}\left(t_{v},c_{\mu_{2}}\cdot \underset{k\in \Omega }{\max}(\left|t_{v}[k]\right|)\right),$$

with

$$t_{v}=\theta \left[\underset{t_{v\;\mathrm{smooth}}}{\underbrace{\sum_{s=0}^{S-1}\psi_{s}*g_{s}-\frac{\lambda_{3}}{\beta_{3}}}}\right]+(1-\theta )\left[\underset{t_{v\;\mathrm{update}}}{\underbrace{v+\frac{\lambda_{4}}{\beta_{4}}}}\right].$$

Note that there is a shrinkage operator with two terms in texture **v**, namely a smoothing term and an updated term, balanced by a parameter *θ*.

### 7.4. The ‘**e**-problem’

**Figure 14.**
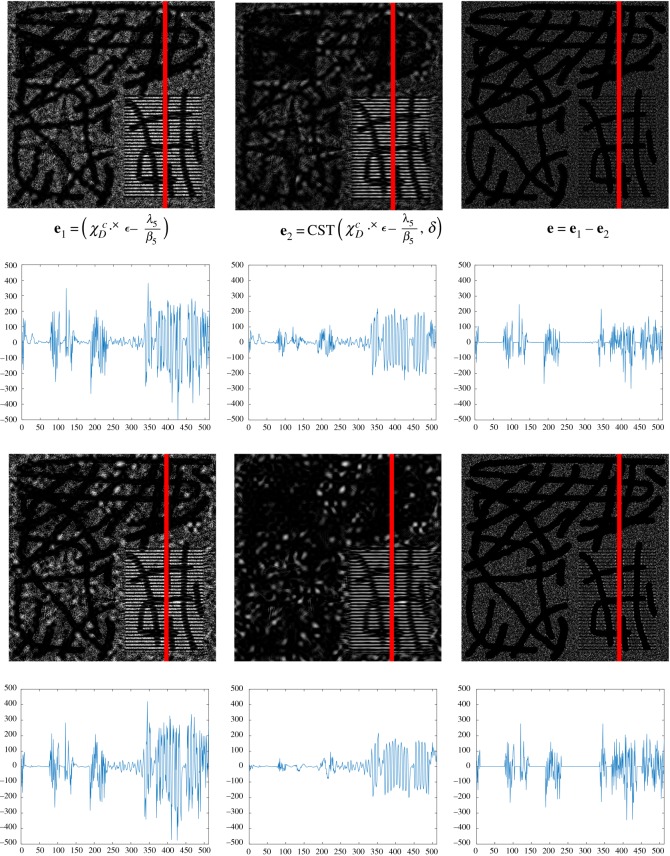
The effect of a noise measurement ${∥C\{\chi_{D}^{c}\cdot^{\times }\epsilon \}∥}_{\ell_{\infty }}$ on *Ω*\*D* in (2.1) by introducing a new variable **e** = *χ*^*c*^_*D*_ · × ***ϵ***. The first and second rows are the images **e**_**1**_, **e**_**2**_ and **e** and their one-dimensional signals along the red lines at iteration 20, respectively. Similarly, the third and fourth rows are at iteration 100. We observe that the cartoon and texture still remain in **e**_**1**_ and its curvelet smoothing term **e**_**2**_ at iteration 20. However, these geometrical signals are reduced in their difference **e** as a result of a subtraction operator. At iteration 100, there is almost no cartoon in **e**_**1**_, **e**_**2**_ and **e**. Note that there is some information in the missing domain *D* due to the updated **λ_5_**.

The noise term ${∥C\{\chi_{D}^{c}\cdot^{\times }\epsilon \}∥}_{\ell_{\infty }}$ in equation (2.1) is suitable to capture high oscillating patterns, especially small-scale types of noise, because of the advantages of the multi-orientation and multi-scale in a curvelet domain and the supremum norm. For a convenient calculation, we introduce a variable **e** as a residual ***ϵ*** in a known domain *Ω*\*D*. The explanation for using the supremum norm of ***ϵ*** in the curvelet domain can be found in (3.4). In particular, there are two terms in (3.4), including a residual in *Ω*\*D* with an updated Lagrange multiplier **λ_5_**, i.e. (*χ*^*c*^_*D*_ · × ***ϵ*** − **λ_5_**/*β*_5_), and its curvelet smoothing term at a level *ν*, i.e. CST(*χ*^*c*^_*D*_ · × ***ϵ*** − **λ_5_**/*β*_5_, *ν*). Assume that, at iteration *t*, there are some remaining signals, e.g. texture, in residual ***ϵ*** in *Ω*\*D*. The curvelet soft-thresholding operator CST( · , · ) reduces noise (or small-scale objects) in (*χ*^*c*^_*D*_ · × ***ϵ*** − **λ_5_**/*β*_5_) at a level *ν* in different scales and orientations. By a subtraction operator in (3.4), **e** at iteration *t* contains mainly noise or small-scale objects (figure 14).

### 7.5. The ‘***ϵ***-problem’

At iteration *t*, we denote the updated residual by the unity condition as

$$\epsilon_{\mathrm{unity}}^{\left(t\right)}=f-u^{\left(t\right)}-v^{\left(t\right)}+\frac{\lambda_{4}^{\left(t-1\right)}}{\beta_{4}},$$

and rewrite (3.6) with the indicator functions on unknown domain *D* and known domain *Ω*\*D* as

$$\epsilon^{\left(t\right)}=\underset{:=\epsilon_{\mathrm{known}}^{\left(t\right)}}{\underbrace{\left[\frac{\beta_{4}}{\beta_{4}+\beta_{5}}\epsilon_{\mathrm{unity}}^{\left(t\right)}+\frac{\beta_{5}}{\beta_{4}+\beta_{5}}\left(e^{\left(t\right)}+\frac{\lambda_{5}^{\left(t-1\right)}}{\beta_{5}}\right)\right]}}\cdot^{\times }\chi_{D}^{c}+\underset{:=\epsilon_{\mathrm{unknown}}^{\left(t\right)}}{\underbrace{\frac{\beta_{4}}{\beta_{4}+\beta_{5}}\epsilon_{\mathrm{unity}}^{\left(t\right)}}}\cdot^{\times }(1-\chi_{D}^{c}).$$

**Figure 15.**
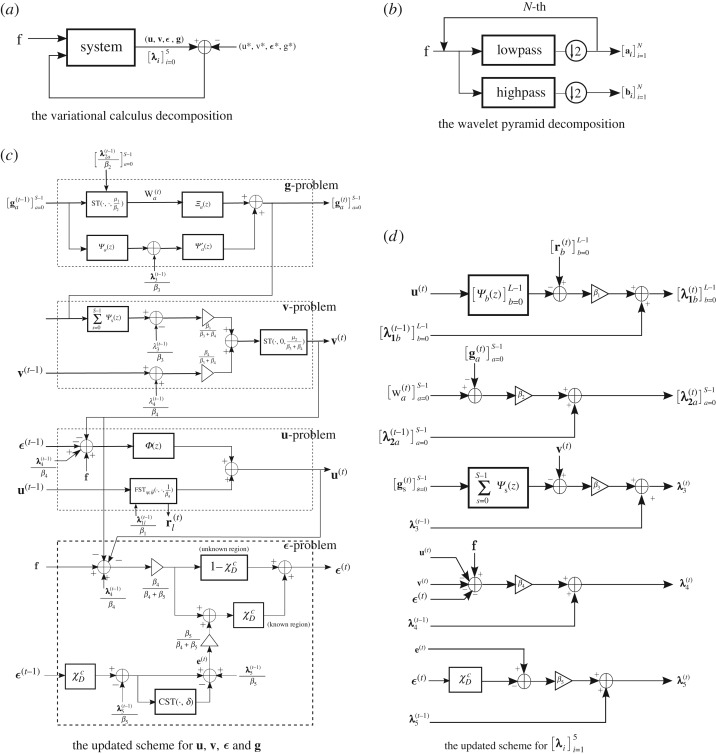
The pyramid scheme for the DG3PD inpainting model.

**Figure 16.**
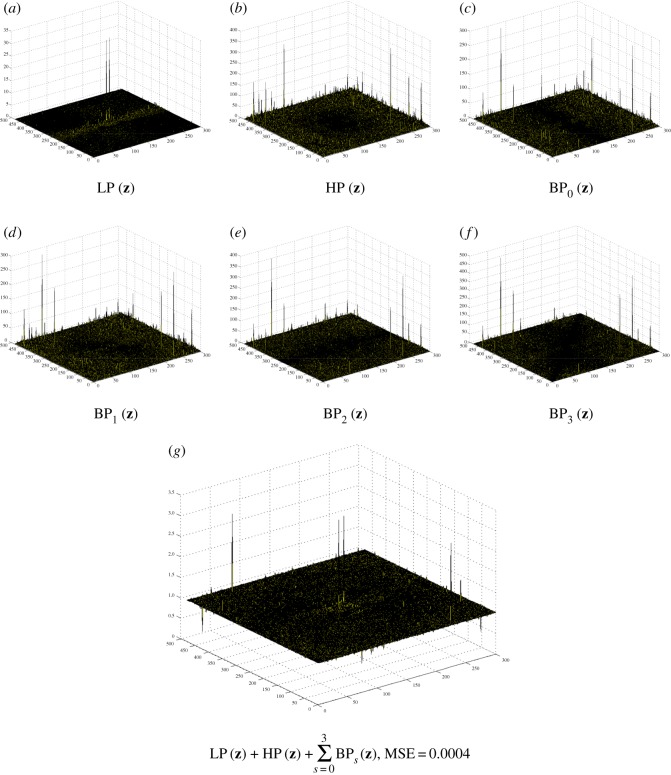
The three-dimensional filters for the fingerprint image in figure 17*a* by DG3PD with the same parameters as in figure 18. The linear filters are different and unique (in terms of computation) to obtain a ‘good’ approximated solution. The scheme of filtering design depends on the characteristics of the image. The variational method will automatically design a filter in a ‘nonlinear way’ (due to minimization via an iterative method) and adapt to the characteristics of each image. MSE, mean squared error.

**Figure 17.**
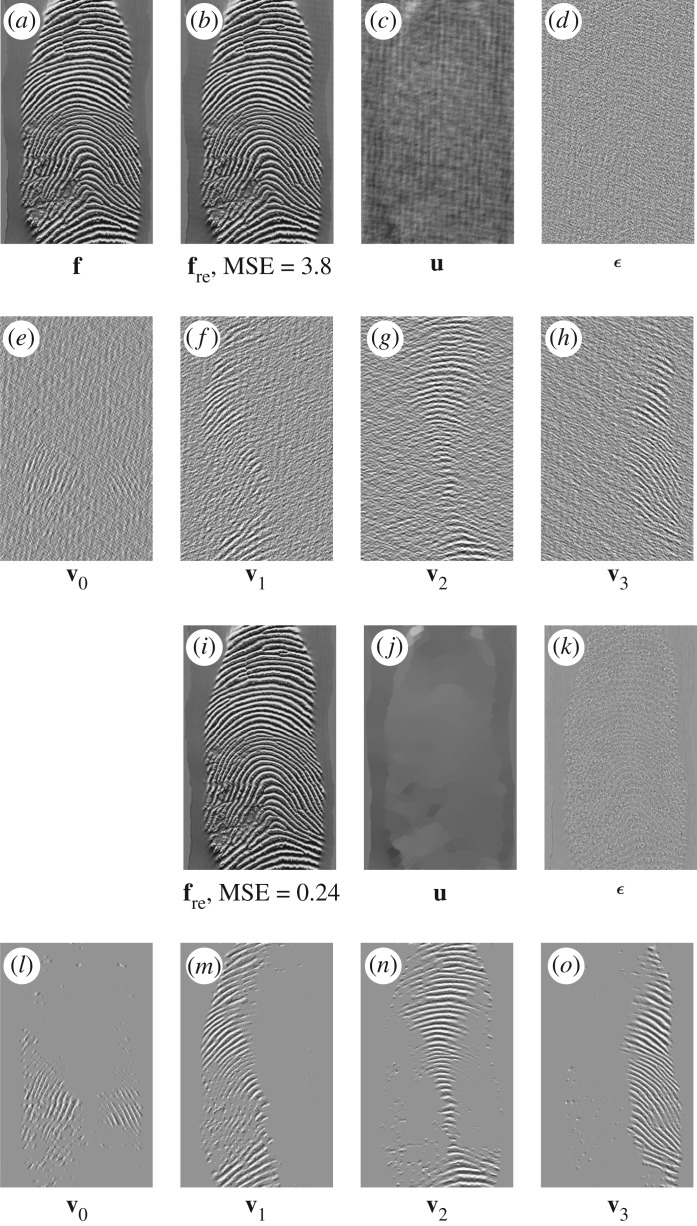
(*a*–*h*) Decomposition by DG3PD applied with the same filters as in figure 19 (in a linear convolution) for a similar fingerprint image to that in figure 18. We observe that applying the same linear filter for a ‘similar’ image cannot give a good result for the decomposition. Thus, the linear filters are ‘unique’ for every image to obtain a suitable decomposition. We apply DG3PD with an iterative method to obtain the decomposition in (*i*–*o*) with the same parameters as in figure 19, and MSE for the reconstructed image (*i*) is much smaller than that in (*b*); see figure 16 for its three-dimensional linear filters.

**Figure 18.**
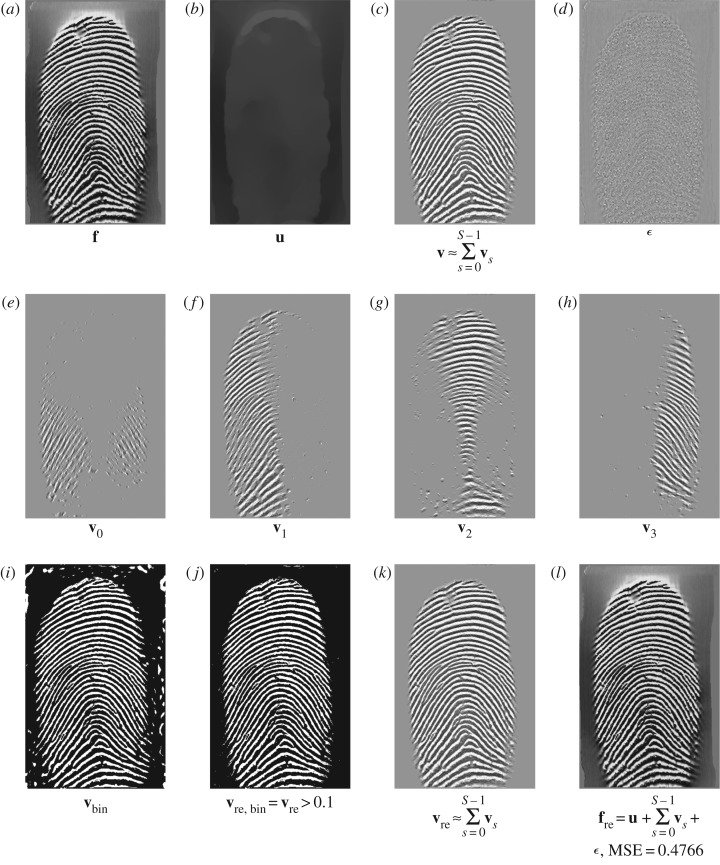
To test the directional property of texture and the piece-wise smooth cartoon in a decomposition, we apply DG3PD [35] for a fingerprint image. Parameters: *β*_4_ = 0.03, *θ* = 0.9, *L* = 100, *S* = 4, *c*_1_ = 1, *c*_2_ = 1.3, *c*_*μ*_1__ = *c*_*μ*_2__ = 0.03, Iter = 200 and *ν* = 15. Cartoon **u** is similar to a lowpass signal and directional texture [**v**_*s*_]^3^_*s*=0_ is similar to directional bandpass signals. Traditionally designing a linear filter is difficult to obtain a smooth lowpass signal with a sharp edge as in (*b*), because it is difficult to define frequencies (i.e. location in the Fourier domain) and their magnitude to approximate a cartoon-like image; see figure 19.

**Figure 19.**
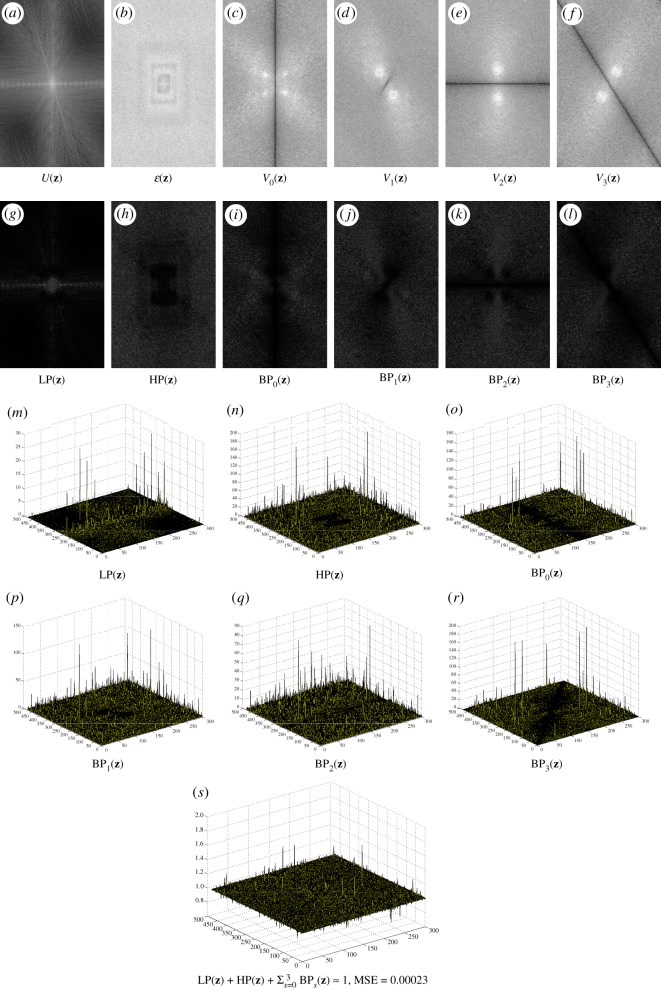
(*a*–*f*) The Fourier spectra of **u**, ***ϵ*** and [**v**_*i*_]^3^_*i*=0_ in figure 18 obtained by DG3PD [35]. Given a spectrum *F*(***z***) of the original image, their corresponding lowpass, highpass and directional bandpass in (*g*–*l*) are defined as LP(z)=U(z)/F(z),HP(z)=E(z)/F(z) and [BP_*s*_(***z***)]^3^_*s*=0_ = [*V*_re*s*_(***z***)]^3^_*s*=0_/*F*(***z***); see (*m*)–(*r*) for their three-dimensional filters. The error in (*s*) is due to the iterative method in ALM. In theory, when the number of iterations goes to infinity, the solution of a decomposition satisfies the condition of the perfect reconstruction. Given *N* = 200 iterations, MSE for the unity condition of all filters is small enough, i.e. it closely satisfies the unity property and the reconstructed signal well approximates the original one.

We see that there are two terms in the residual ***ϵ***^(*t*)^, including the updated term ***ϵ***^(*t*)^_known_ for *Ω*\*D* and ***ϵ***^(*t*)^_unknown_ for *D*. Also, there are two other terms for updating ***ϵ***^(*t*)^_known_ in *Ω*\*D*, namely ***ϵ***^(*t*)^_unity_ (from a unity condition) and **e**^(*t*)^ (from a curvelet smoothing operator). In contrast to an open loop in a pyramidal decomposition (Laplacian pyramid, wavelet, curvelet, etc.), the solution of DG3PD inpainting can be considered as a closed loop of the pyramid scheme with the true solution (u*, v*, ϵ*, g*) in the minimization problem. The loop of the updated Lagrange multipliers can be seen as a refinement. If we remove this loop from the scheme in figure 15*a*, the minimization in DG3PD becomes the quadratic penalty method. The removal would cause an imperfect reconstruction for the constraints, e.g. **u** + **v** + ***ϵ***≠**f**; see fig. 7(p) and (q) in [35]. We note that the lowpass, bandpass and highpass filters for the cartoon, texture and residual components are ‘custom-made’ for each image, resulting in different filters for different images (figures 16 and 17). The directional properties of texture and their directional filter banks are illustrated in figures 18 and 19.

## 8. Conclusion

In this paper, we have addressed the very challenging task of restoring images with a large number of missing pixels, whose existing pixels are corrupted by noise and, importantly, the image to be restored contains both cartoon and texture elements. The proposed DG3PD model for inpainting and denoising can cope with this threefold difficult problem. The task of simultaneous inpainting and denoising for cartoon and texture components is solved by DG3PD decomposition, followed by inpainting and denoising both components separately and, finally, image restoration by synthesis of the restored components. More specifically, the DG3PD inpainting model is based on a regularization in the Banach space in a discrete setting which is solved by the augmented Lagrangian method and the alternating direction method of multipliers.

In summary, the decomposition step of the proposed method has two major advantages for tackling this challenging problem. Firstly, the decomposition step denoises simultaneously the cartoon and texture component. Secondly, it allows the cartoon and texture to be handled in different ways. The cartoon image is inpainted by DG3PD, as described in §§2 and 3. Separately, the texture component is inpainted followed by further denoising as described in §4. Therefore, the proposed DG3PD decomposition can also be understood as breaking the full problem down into ‘smaller’ subproblems (e.g. texture only inpainting) which are easier to solve.

Image restoration (or image denoising and inpainting) can be understood as an inverse problem and it can be described by a discrete innovation model (figure 10). The assumption in this procedure is that signal has a sparse representation in suitable transform domains. It is known from probability theory that the sparsity (by ℓ_1_) is connected to the Laplace distribution of the signal, which results in the heavy tail distribution. Note that the Laplace distribution with the ℓ_1_ norm is one of the best approximations of the sparsity by the ℓ_0_ norm.

Moreover, by choosing the priors and posterior according to the Bayesian framework and an MAP, we can understand the selection of parameters (see figure 9 for the results of the selection of the heavy-tailed distribution, e.g. the Laplace distribution). Note that the MAP requires an assumption on the noise, e.g. Gaussian or Laplacian, to establish a minimization. In our proposed model, there is no requirement for an assumption on noise distribution, e.g. independent identical distributed, or correlated and Gaussian or non-Gaussian (due to the measurement ${∥C\{\cdot \}∥}_{\ell_{\infty }}$, which is similar to the Dantzig selector [60]).

## Data accessibility

Data are available from https://figshare.com/s/76d30e70e6e131da2a08.
